# A toxic gain-of-function mechanism in C9orf72 ALS impairs the autophagy-lysosome pathway in neurons

**DOI:** 10.1186/s40478-023-01648-0

**Published:** 2023-09-18

**Authors:** Jimmy Beckers, Arun Kumar Tharkeshwar, Laura Fumagalli, Matilde Contardo, Evelien Van Schoor, Raheem Fazal, Dietmar Rudolf Thal, Siddharthan Chandran, Renzo Mancuso, Ludo Van Den Bosch, Philip Van Damme

**Affiliations:** 1 Department of Neurosciences, Experimental Neurology and Leuven Brain Institute (LBI), KU Louvain – University of Leuven, Leuven, Belgium; 2 Center for Brain and Disease Research, Laboratory of Neurobiology, VIB, Leuven, Belgium; 3 Department of Human Genetics, KU Leuven, Louvain, Belgium; 4 Center for Molecular Neurology, Microglia and Inflammation in Neurological Disorders (MIND) Lab, VIB, Antwerp, Belgium; 5 Department of Biomedical Sciences, University of Antwerp, Antwerp, Belgium; 6 Laboratory of Neuropathology, Department of Imaging and Pathology, Leuven Brain Institute (LBI), KU Louvain – University of Leuven, Leuven, Belgium; 7 Department of Pathology, University Hospitals Leuven, Louvain, Belgium; 8 UK Dementia Research Institute, University of Edinburgh, Edinburgh, EH16 4SB UK; 9 Centre for Clinical Brain Sciences, University of Edinburgh, Edinburgh, EH16 4SB UK; 10 Department of Neurology, University Hospitals Leuven, Louvain, Belgium

**Keywords:** Amyotrophic lateral sclerosis, Autophagy, *C9orf72*, Endosome, Human iPSC-derived spinal motor neurons, Lysosome, p62, TBK1

## Abstract

**Background:**

Motor neurons (MNs), which are primarily affected in amyotrophic lateral sclerosis (ALS), are a specialized type of neurons that are long and non-dividing. Given their unique structure, these cells heavily rely on transport of organelles along their axons and the process of autophagy to maintain their cellular homeostasis. It has been shown that disruption of the autophagy pathway is sufficient to cause progressive neurodegeneration and defects in autophagy have been associated with various subtypes of ALS, including those caused by hexanucleotide repeat expansions in the *C9orf72* gene. A more comprehensive understanding of the dysfunctional cellular mechanisms will help rationalize the design of potent and selective therapies for *C9orf72*-ALS.

**Methods:**

In this study, we used induced pluripotent stem cell (iPSC)-derived MNs from *C9orf72*-ALS patients and isogenic control lines to identify the underlying mechanisms causing dysregulations of the autophagy-lysosome pathway. Additionally, to ascertain the potential impact of *C9orf72* loss-of-function on autophagic defects, we characterized the observed phenotypes in a *C9orf72* knockout iPSC line (C9-KO).

**Results:**

Despite the evident presence of dysfunctions in several aspects of the autophagy-lysosome pathway, such as disrupted lysosomal homeostasis, abnormal lysosome morphology, inhibition of autophagic flux, and accumulation of p62 in *C9orf72*-ALS MNs, we were surprised to find that *C9orf72* loss-of-function had minimal influence on these phenotypes. Instead, we primarily observed impairment in endosome maturation as a result of *C9orf72* loss-of-function. Additionally, our study shed light on the pathological mechanisms underlying *C9orf72*-ALS, as we detected an increased TBK1 phosphorylation at S172 in MNs derived from *C9orf72* ALS patients.

**Conclusions:**

Our data provides further insight into the involvement of defects in the autophagy-lysosome pathway in *C9orf72*-ALS and strongly indicate that those defects are mainly due to the toxic gain-of-function mechanisms underlying *C9orf72*-ALS.

**Supplementary Information:**

The online version contains supplementary material available at 10.1186/s40478-023-01648-0.

## Introduction

Amyotrophic lateral sclerosis (ALS) is a devastating neurodegenerative disorder characterized by the progressive and rapid loss of both upper and lower motor neurons (MNs) in the motor cortex, brainstem, and spinal cord. It is primarily an adult-onset, incurable disease, ultimately leading to muscle weakness, atrophy, and a limited survival period of 2–5 years after symptom onset, mainly due to respiratory failure and the lack of effective treatment options [1]. While the majority of ALS cases (~ 90%) have no affected family members and are thus classified as sporadic ALS (sALS), the other 10% of familial ALS (fALS) patients have a hereditary component within affected families. Despite being clinically indistinguishable, both sALS and fALS types exhibit similar neuropathological features, including loss of neurons, cytoplasmic mislocalization of TAR DNA binding protein 43 (TDP-43) and the formation of insoluble protein aggregates containing TDP-43 [1–4]. In 2011, a GGGGCC (G4C2) hexanucleotide repeat expansion (HRE) in the 5’ non-coding sequence of the *C9orf72* gene was identified as the most common genetic cause of both fALS and sALS [5, 6]. Notably, this HRE in *C9orf72* is also one of the primary genetic cause of frontotemporal dementia (FTD), the second most common form of early-onset dementia in individuals under 65 years, after Alzheimer’s disease (AD) [5, 6]. Interestingly, ALS and FTD are the extremes of the same disease spectrum having several shared phenotypes such as the deposition of TDP-43 aggregates which suggests that an effective treatment for ALS might potentially also be beneficial for patients suffering from FTD [7–10].

Substantial progress has been made in understanding the underlying pathogenic mechanisms of C9-ALS/FTD. Three distinct non-mutually exclusive mechanisms have been proposed: a loss-of-function mechanism characterized by reduced C9orf72 protein levels, known as C9orf72 haploinsufficiency [11–13]; toxic gain-of-function mechanisms where the HRE leads to the accumulation of sense and antisense RNAs within the nucleus as foci [6, 11, 14, 15] or undergo repeat-associated non-ATG (RAN) translation to produce toxic dipeptide-repeat proteins (DPRs) [11, 16–18]. The research done in the last decade rather points towards toxic gain-of-function mechanisms as drivers of the disease, which are potentially aggravated by C9orf72 loss-of-function [19–22].

SQSTM1/p62, an autophagy receptor protein, is commonly found in TDP-43 aggregates, indicating a dysfunction in proteostasis pathways such as the autophagy [23, 24]. Interestingly, several ALS-FTD-related genes, including *ubiquilin 2* (*UBQLN2*), *valosin containing protein* (*VCP*), *optineurin* (*OPTN*) and *TANK binding kinase 1* (*TBK1*), are implicated in the autophagy-lysosome pathway [8, 25]. In addition, disruptions in lysosomal homeostasis have been associated with multiple neurodegenerative diseases, including FTD and ALS [26–30]. Additionally, autophagic abnormalities have been reported in multiple ALS subtypes [31], and ablation of several core autophagy genes has been shown to induce progressive neuronal degeneration, implying the indispensability of the pathway for neuronal health [32, 33]. Interestingly, both *C9orf72* haploinsufficiency and *C9orf72* HRE toxic gain-of-function mechanisms are thought to disrupt the autophagy pathway, contributing to disease progression in C9-ALS/FTD [19].

To gain deeper insights into the impact of autophagic defects on MN degeneration in the context of C9-ALS/FTD, we used patient-derived hiPSC-derivedMNs and CRISPR/Cas9-mediated genome editing technology to create a *C9orf72* knockout hiPSC line, allowing us to assess the functional consequences of reduced C9orf72 protein levels on the autophagy-lysosome pathway. Our findings collectively reveal that *C9orf72* HRE toxic gain-of-function leads to defects in multiple aspects of the autophagy-lysosome pathway, accompanied by increased TBK1 phosphorylation. However, our data also provides additional evidence that questions the direct involvement of *C9orf72* haploinsufficiency in autophagic defects, as knockout of *C9orf72* primarily resulted in deficiencies in endosome maturation that did not significantly impact autophagy in hiPSC-derived MNs.

## Results

### *C9orf72* MNs display impaired lysosomal transport

**Fig. 1:**
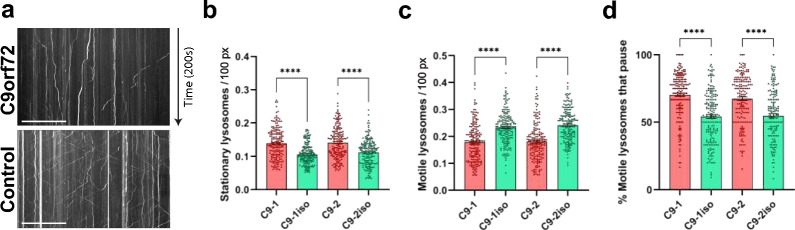
*C9orf72* MNs display impairment of lysosomal trafficking along neurites. **a** Example kymograph from 40-day-old C9orf72 and isogenic control MNs loaded with Lysotracker Red. Tilted and straight vertical lines represent moving and stationary lysosomes respectively. Scale bar, 30 µM. **b** Quantification of the number of stationary lysosomes in proportion to neurite length (in pixels, px). **c** Quantification of the number of motile lysosomes in proportion to neurite length (in pixels, px). **d** Quantification of the percentage of motile lysosomes that pause or stop during their transport. Data represent mean ± SEM; data are pooled from three independent differentiations, and each dot represents a neurite (n = 180 neurites for all conditions). Statistical significance was assessed by one-way ANOVA and Tukey’s multiple comparison tests (**b–d**); *p < 0.05, **p < 0.01, ***p < 0.001, ****p < 0.0001

Axonal transport defects play a crucial role in the disease mechanism of several neurodegenerative diseases, including ALS/FTD. Several genes associated with ALS, such as the *C9orf72* HRE, have been implicated in this process, and these defects have been observed in various disease models [34–39]. Autophagy, another crucial cell homeostasis pathway frequently stated in ALS pathology, also heavily relies on axonal transport for both the delivery of degradative lysosomes to more distal parts of the axon and for the retrograde transport of autophagic vesicles [19, 31, 40]. Therefore, we used two pairs of *C9orf72* patient iPSC-derived MNs and their isogenic controls previously described in [41] and evaluated the movement of lysosomes and autolysosomes through live imaging of *C9orf72* and isogenic control iPSC-derived MNs stained with the acidotropic dye Lysotracker. We followed a well-established protocol to differentiate patient and isogenic control iPSCs into MNs [34–36, 42, 43] (Additional file 1: Fig. S1a). Immunostaining with specific markers for MNs, including ISL1, ChAT, and SMI-32, confirmed that both the patient *C9orf72* lines and their isogenic control lines generated MNs with a differentiation efficiency ranging from 81 to 90% (Additional file 1: Fig. S1b–f). Kymograph-based analysis was used to analyze the transport of (auto)lysosomes labeled with Lysotracker Red along the neurites of MNs (Fig. 1a). Our findings revealed a significant increase in the proportion of stationary (auto)lysosomes (Fig. 1b) and a notable decrease in both the proportion of moving (auto)lysosomes (Fig. 1c) and the proportion of motile (auto)lysosomes that paused or halted during axonal transport (Fig. 1d) in mutant C9orf72 MNs compared to controls. Collectively, these observations align with previous studies demonstrating lysosomal transport defects in *C9orf72* iPSC-derived MNs [37, 38].

### *C9orf72* MNs have fewer and enlarged lysosomes

**Fig. 2:**
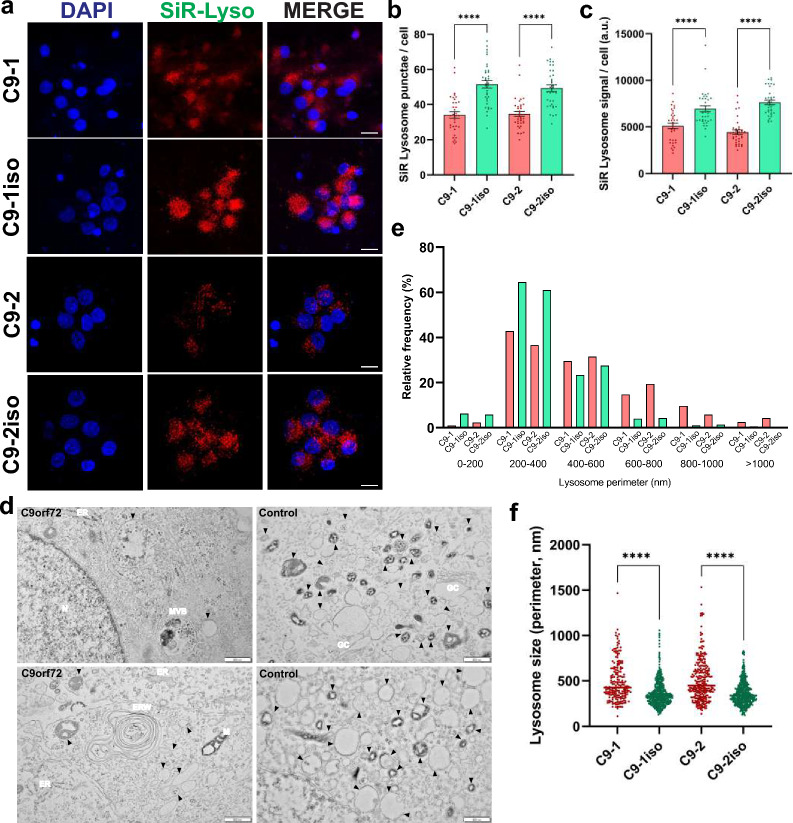
*C9orf72* MNs have reduced lysosome numbers and altered lysosome morphology. **a** Fluorescent microscopy images of 40-day-old C9orf72 and isogenic control MNs labeled with SiR-Lysosome (SiR-Lyso), a dye that selectively labels Cathepsin D-positive lysosomes. Scale bar = 50 µm. **b, c** Quantification of the SiR-Lysosome puncta number (**b**) and fluorescent intensity per cell (**c**) from the images shown in (**a**); each dot represents one confocal image that was analyzed (n = 35 for all conditions). **d** Representative TEM images from 40-day old MNs used to quantify the size of lysosomes/late endosome (indicated by black arrowheads). Multivesicular bodies (MVB), Mitochondria (M), Golgi complex (GC), the endoplasmic reticulum (ER) and the ER whorls (ERW) are marked on the images. **e**, **f** Relative frequency distribution (**e**) and quantification (**f**) of the lysosomal circumference measured from TEM images as shown in (**d**); each dot represents a lysosome that was measured (C9-1, n = 197; C9-1iso, n = 472; C9-2, n = 257; C9-2iso, n = 445). Data represent mean ± SEM; data are pooled from three independent differentiations. Statistical significance was assessed by one-way ANOVA and Tukey’s multiple comparison tests (**b, c**) or Kruskal–Wallis test and Dunn’s multiple comparison tests (**f**); *p < 0.05, **p < 0.01, ***p < 0.001, ****p < 0.0001

The proper functioning of neuronal lysosomes relies heavily on both anterograde axonal transport for delivering endosomes and lysosomes to distal axonal regions and retrograde axonal transport crucial for the maturation of autophagic vesicles and (endo)lysosomes [44, 45]. Interestingly, flow cytometry analysis using Lysotracker Red revealed a general decrease of (auto)lysosomal vesicles in *C9orf72* MNs (Additional file 1: Fig. S2a, b). To determine whether the observed axonal transport defects of acidic vesicles affect neuronal lysosome homeostasis, we also utilized SiR-Lysosome, a probe that stains mature lysosomes that contain active cathepsin D (CTSD) (Fig. 2a). Our results showed a decrease in the number of SiR-Lysosome-positive puncta in *C9orf72* MNs (Fig. 2b, c). Furthermore, we employed DQ-Red BSA dye, which allows to assess both endolysosomal activity and lysosomal cargo delivery as this self-quenched dye is taken up via endocytosis and targeted to the lysosomes, where it is enzymatically cleaved and becomes fluorescent [46–48]. Consistent with the reduction in Lysotracker Red fluorescence, fluorescence intensities upon enzymatic cleavage of DQ-Red BSA were reduced by ~ 25% relative to isogenic control cells (Additional file 1: Fig. S2c, d). Moreover, analysis of iPSC-derived MN lysates using Western blot (Additional file 1: Fig. S2e, f) and a fluorescence-based CTSD activity assay (Additional file 1: Fig. S2g) revealed a reduction in mature CTSD protein levels and relative levels of CTSD activity respectively in *C9orf72* lysates compared to controls. Of note, since these assays are only semi-quantitative, we are unable to discern between a reduced enzymatic activity of CTSD or an overall reduction in mature CTSD protein levels as a cause for reduced CTSD activity levels although our data points towards the latter explanation.

Next, we investigated endolysosomal morphology in iPSC-derived *C9orf72* and control MNs using transmission electron microscopy (TEM) and discovered an increased proportion of enlarged lysosomes, as well as multivesicular bodies (MVB) and autophagic ER whorls, indicative of both autophagy and ER stress [49, 50] (Fig. 2d–f). These findings highlight that the *C9orf72* HRE leads to alterations in lysosomal homeostasis.

### C9orf72 MNs exhibit TDP-43 pathology and reduced cell viability upon aging

**Fig. 3:**
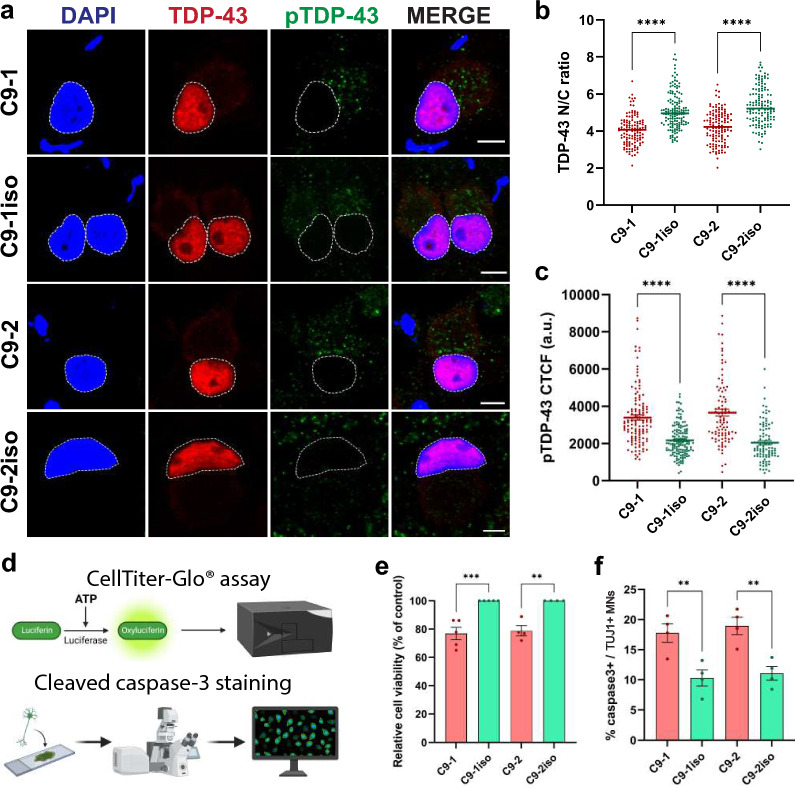
Upon aging, *C9orf72* MNs display mislocalization of TDP-43, increased phosphorylation of TDP-43 and a reduced cell viability. **a** Immunocytochemistry (ICC) of aged, 60-day-old C9orf72 and isogenic control MNs stained for TDP-43 and phosphorylated TDP-43 (pTDP-43). The presence of a typical neuronal morphology (which includes a soma and elongated neurites was used to select the MNs for analysis. Scale bar = 10 µm. **b**, **c** Quantifications of the nuclear vs cytoplasmic (N/C) TDP-43 ratio (**b**) and the corrected total cell fluorescence (CTCF) of pTDP-43 (**c**) from the ICC images shown in (**a**). Each dot represents a cell that was measured (C9-1, n = 124; C9-1iso, n = 145; C9-2, n = 133; C9-2iso, n = 132). **d** Schematic overview of the two methods used to measure cell viability. **e** Quantification of the relative cell viability in 60-day-old C9orf72 MNs relative to their isogenic controls as measured by the CellTiter-Glo® assay. For each independent differentiation, at least 6 technical replicates of 5000 cells were plated in 96-well plates the average intensity of each C9orf72 patient line was normalized to that of their respective isogenic control; each dot represents one biological replicate. **f** Quantification of the percentage of apoptotic cells staining positive for cleaved caspase-3 in the TUJ1-positive 60-day-old MN population. Each dot represents one biological replicate in which between 126 and 239 TUJ1-positive cells were scored for cleaved caspase-3 staining. Data represent mean ± SEM; data are pooled from four-five independent differentiations. Statistical significance was assessed by one-way ANOVA and Tukey’s multiple comparison test (**e, f**) or Kruskal–Wallis test and Dunn’s multiple comparison test (**b, c**); *p < 0.05, **p < 0.01, ***p < 0.001, ****p < 0.0001

During the last decade, multiple groups successfully detected RNA foci and some DPRs using patient iPSC-derived neuronal models [37, 38, 41, 51–55]. However, the two major hallmarks of ALS—cytoplasmic mislocalization of TDP-43 and MN degeneration were not consistently observed, casting some doubt on the relevance of these model systems [56]. In our initial assessment of the subcellular distribution of TDP-43 in 40-day-old patient iPSC-derived MNs and isogenic controls, we did not observe any mislocalization of the protein (Additional file 1: Fig. S3a, b). Additionally, when assessing the disease-associated S409/410 phosphorylation of TDP-43 [57], we only detected a slight increase in its phosphorylation levels (Additional file 1: Fig. S3a, c). However, as the MN cultures aged, we observed a subtle cytoplasmic mislocalization of TDP-43 (Fig. 3a, b). Interestingly, this age-dependent decrease in nuclear/cytoplasmic ratio of TDP-43 coincided with a significant increase in the abnormal S409/410 phosphorylation of TDP-43 (Fig. 3a, c).

Next, to investigate MN degeneration/survival, we employed two different approaches. First, we used the CellTiter-Glo® assay to measure metabolically active cells based on the quantification of cellular ATP levels (Fig. 3d, top panel). Second, we performed immunostaining for cleaved caspase-3 in MN cultures to estimate the percentage of apoptotic cells (Fig. 3d, bottom panel). Interestingly, in 40-day-old MNs we only detected a minor decrease in metabolically active cells (Additional file 1: Fig. S3d) and minimal evidence of apoptosis (Additional file 1: Fig. S3e). In contrast, the TDP-43 pathology that became noticeable upon aging in 60-day old *C9orf72* MNs coincided with a reduction in cell viability (Fig. 3e) as well as with increased levels of cleaved caspase-3 (Fig. 3f). Taken together, our findings demonstrate that TDP-43 becomes mislocalized to the cytoplasm and MN cell viability declines as the cells age.

### *C9orf72* MNs show protein aggregates, insoluble full-length, and C-terminal fragments of TDP-43

**Fig. 4:**
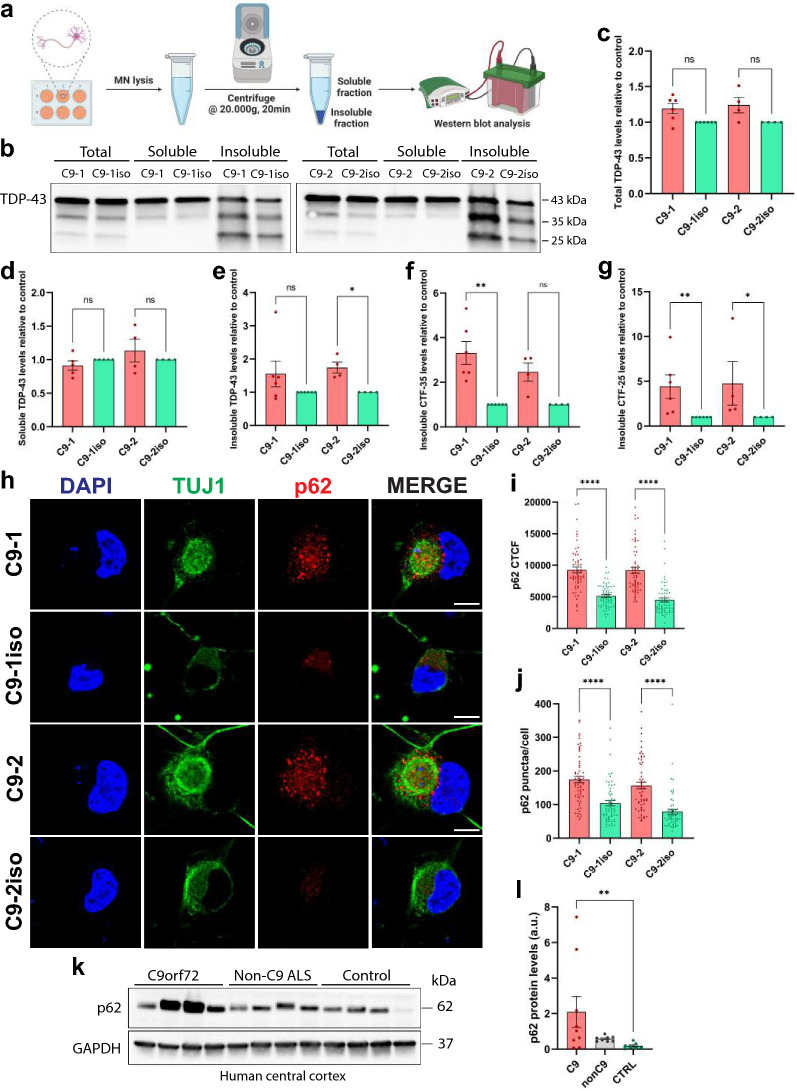
Presence of the *C9orf72* HRE results in increased levels of insoluble full-length and C-terminal fragments of TDP-43 and an increase in p62 levels. **a** Schematic representation of the worklow used to separate soluble and insoluble fractions from iPSC-derived MNs. **b** Representative Western blot showing TDP-43 in total (unfractionated), soluble and insoluble fractions. **c–g** Quantifications of (**b**), measuring the levels of full-length TDP-43 in the total fraction (**c**), full-length TDP-43 in the soluble fraction (**d**), full-length TDP-43 in the insoluble fraction (**e**), the c-terminal fragment of 35 kDa (CTF-35) in the insoluble fraction (**f**) and the c-terminal fragment of 25 kDa (CTF-25) in the insoluble fraction (**g**). Each dot represents one biological replicate. **h** Immunocytochemistry (ICC) images of 40-day-old C9orf72 and isogenic control iPSC-derived MNs stained for the aggregate marker p62 and a neuronal marker TUJ1. Scale bar = 10 µm. **i, j** Quantifications of the corrected total cell fluorescence (CTCF) (**i**) and p62 puncta number per cell (**j**) from the ICC images shown in (**h**); each dot represents a confocal image containing one single MN that was analyzed (n = 60 for all conditions). **k** Representative Western blot detecting p62 in *post-mortem* human central cortex tissue lysates from C9orf72 ALS patients (C9orf72), ALS patients tested negative for the C9orf72 HRE (Non-C9 ALS) and healthy controls (Control). **l**, Quantification of (**k**), measuring p62 protein levels; each dot represents one tissue sample (C9orf72, n = 9; Non-C9 ALS, n = 8; Control, n = 7). GAPDH was used to normalize data. Data represent mean ± SEM; data are pooled from three independent differentiations. Statistical significance was assessed by one-way ANOVA and Tukey’s multiple comparison test (**i, j**) or Kruskal–Wallis test and Dunn’s multiple comparison test (**c–g, l**); *p < 0.05, **p < 0.01, ***p < 0.001, ****p < 0.0001

Confocal microscopic observations did not reveal apparent aggregates that stained positive for pTDP-43 or TDP-43, which are major indicators of ALS pathology. However, a previously established protein fractionation protocol (Fig. 4a) revealed increased levels of both insoluble full-length and C-terminal TDP-43 fragments (CTFs) measuring 35 and 25kDa resulting from proteolytic cleavage in mutant TDP-43 iPSC-derived MNs [34, 58]. While these insoluble TDP-43 proteins may represent early stages (seeds) of TDP-43 aggregates, the presence of insoluble TDP-43 CTFs is also considered to be a hallmark of ALS [59]. Similar to the observations in *TDP-43* iPSC-derived MNs [34], *C9orf72* iPSC-derived MNs exhibited increased levels of insoluble TDP-43 and TDP-43 CTFs (Fig. 4b, e–g) despite the absence of larger TDP-43 aggregates. Notably, total levels of TDP-43, as well as soluble TDP-43 levels, remained unchanged in *C9orf72* MNs, indicating no major problems with protein stability or autoregulation (Fig. 4c, d).

**Fig. 5:**
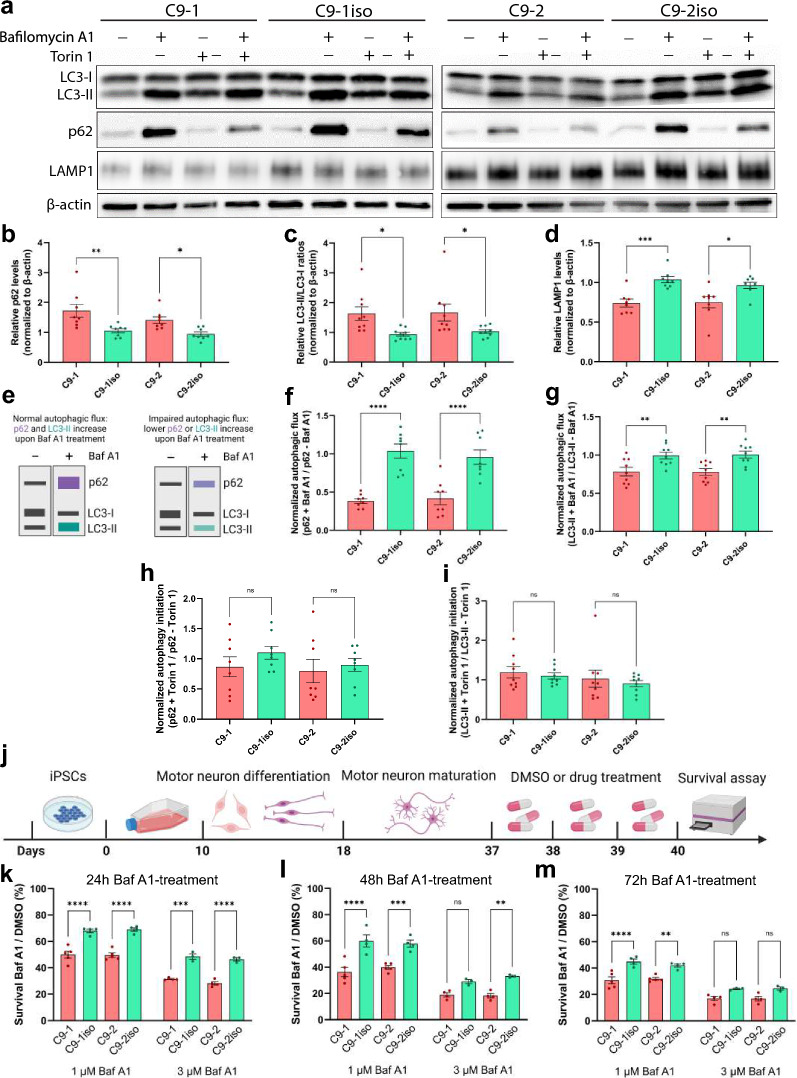
The autophagic pathway is impaired in *C9orf72* MNs. **a,** Representative Western blot of iPSC-derived MNs treated with either DMSO, the autophagy inhibitor Bafilomycin A1 (Baf A1), the autophagy inducer Torin 1 or a combination of the latter, immunostained for LC3-I and LC3-II, p62, LAMP1 and β-actin, used to measure levels of autophagosomes, aggregates, lysosomes or for protein normalization respectively. **b,c,d,** Quantifications of the Western blot shown in (**a**), measuring the relative levels of p62 (**b**), the ratio of LC3-II/LC3-I (**c**) and LAMP1 (**d**); each dot represents one biological replicate. **e** Schematic representation of the autophagic flux assay. By inhibiting the autophagy pathway with high doses of Baf A1 and measuring the concomitant increase in LC3-II or p62, we get an estimate of the amount of LC3-II or p62 that would have been degraded when no drugs were added (= autophagic flux). **f**, **g** Quantifications of the Western blot shown in (**a**), measuring the levels of p62 (**f**) or LC3-II (**g**) after treatment with Baf A1 and dividing those with the levels of either p62 or LC3-II without treatment; each dot represents one biological replicate. **h,i,** Quantifications of the Western blot shown in (**a**), measuring the relative levels op p62 (**h**), LC3-II (**i**) after treatment with Torin 1 and dividing those with the levels of either p62 or LC3-II without treatment; each dot represents one biological replicate. **j** Experimental outline of the Baf A1 vulnerability assay in iPSC-derived MNs. Following normal MN differentiation, DMSO, 1 µM or 3 µM of Baf A1 are added to the medium at day 37, 38 or 39. At day 40, the CellTiter-Glo® assay was used to asses cell survival. **k, l, m,** Quantification of the survival rate after 24 h (**k**), 48 h (**l**) or 72 h (**m**) treatment with Baf A1. The survival rate of iPSC-derived MNs is calculated by normalizing the CellTiter-Glo® signal of Baf A1-treated cells to DMSO control-treated wells; each dot represents the average of one biological replicate. Data represent mean ± SEM; data are pooled from four-five (**k–m**) or eight-nine (**b–d**, **f–i**) independent differentiations. Statistical significance was assessed by one-way ANOVA and Tukey’s multiple comparison test (**b–d**, **f–i**, **k–m**); *p < 0.05, **p < 0.01, ***p < 0.001, ****p < 0.0001, ns = not significant

Another approach to assess premature protein aggregation involves examining the levels of SQSTM1/p62. p62 plays a multifaceted role in cellular proteostasis as it is involved in different signal transduction pathways [60, 61]. However, p62 is best known for its role as an autophagy receptor for ubiquitinated proteins, and increased p62 levels often indicate reduced aggregate clearance by the autophagy system [60, 62]. Interestingly, elevated levels of p62 are frequently observed in *post-mortem* tissue from ALS patients [63–65], and increased p62 levels have been found in iPSC-derived cortical neurons and MNs of *C9orf72* patients [51, 52]. By measuring basal endogenous p62 using immunofluorescence (Fig. 4h), we could replicate the increase in both total p62 signal and p62 puncta numbers per MN (Fig. 4i, j). Notably, elevated p62 levels were also detected in 40-day-old *C9orf72* iPSC-derived MN lysates using Western blot, thus preceding TDP-43 mislocalization and cell death (Fig. 5a, b). Furthermore, analysis of *post-mortem* spinal cord and central cortex tissue from *C9orf72* carriers also showed increased levels of p62 (Fig. 4k, l; Additional file 1: Fig. S4d, g). More detailed investigation of these *C9orf72 post-mortem* brain and spinal cord tissue lysates uncovered additional indications of dysregulation in the autophagy-lysosome pathway, as evidenced by alterations in the levels of LAMP1 and the ratio of LC3-II/LC3-I (Additional file 1: Fig. S4a–f). These findings demonstrate that by using *C9orf72* iPSC-derived MNs, we were able to replicate early signs of TDP-43 pathology and protein aggregation observed in *post-mortem* material from *C9orf72* patients.

### Autophagy is impaired in *C9orf72* MNs

To further support our findings, we treated mature *C9orf72* and control iPSC-derived MNs with either Bafilomycin A1 (Baf A1), a potent inhibitor of the vacuolar-type H + -ATPase, Torin 1, a strong mTOR inhibitor or a combination of both for a period of 24 h (Fig. 5a). Baf A1 was used to block the fusion of lysosomes with autophagosomes, leading to autophagy inhibition, while Torin 1 induced autophagy by inhibiting mTOR. When we initially quantified the LC3-II/LC3-I ratio in DMSO-treated cells we observed an increase in the ratio of LC3-II/LC3-I in patient MNs (Fig. 5c). Interestingly, in line with the reduced lysosome count, untreated *C9orf72* iPSC-derived MNs exhibited a significant decrease in basal levels of LAMP1 (Fig. 5d). In addition, to assess autophagic flux, we blocked the autophagy pathway using high doses of Baf A1 and measured the resulting increase in LC3-II and p62 levels, providing an estimate of the amount of LC3-II or p62 that would have been degraded in the absence of drugs (Fig. 5e). By dividing the levels of p62 or LC3-II in Baf A1-treated cells by the respective levels in untreated cells, we observed a significant reduction of autophagic flux (Fig. 5f, g). Despite this autophagic flux dysregulation, immunostaining of autophagy markers in *C9orf72* and control iPSC-derived MNs treated with Torin 1 normalized to untreated cells revealed no significant defects in autophagy initiation (Fig. 5h-j). Additionally, we assessed MN survival during autophagic stress by treating mature *C9orf72* and control iPSC-derived MNs with Baf A1 (Fig. 5j. Both genotypes displayed a dose- and time-dependent reduction in MN survival (Fig. 5j–m). In nearly all treatment conditions, the viability of *C9orf72* MNs was significantly reduced compared to the respective treated isogenic control cells (Fig. 5k–m). To validate these findings, we used Lys05, a novel and potent autophagy pathway inhibitor, and obtained similar results (Additional file 1: Fig. S8a) [66]. In conclusion, these observations provide compelling evidence of diminished autophagic flux and increased vulnerability to lysosomal stress in *C9orf72* iPSC-derived MNs.

### C9orf72 protein levels are reduced in *C9orf72* ALS patients, but this haploinsufficiency is not replicated in iPSC-derived MNs

At present, the impact of C9orf72 haploinsufficiency in *C9orf72* ALS/FTD is still a subject of debate, as multiple studies using different model systems have reported conflicting results [13, 19, 67]. We used a very specific C9orf72 antibody [68] to assess C9orf72 protein levels in human *post-mortem* and iPSC-derived MN samples from *C9orf72* patients and isogenic controls using Western blot (Additional file 1: Fig. S5a–c). While a marked decrease in C9orf72 protein levels was observed in *post-mortem* central cortex and spinal cord samples (Additional file 1: Fig. S5d, e), we did not observe C9orf72 haploinsufficiency in iPSC-derived MN samples derived from two *C9orf72* patients when compared to their respective isogenic controls (Additional file 1: Fig. S5f). These findings once again highlight the challenges associated with investigating and modeling the role of C9orf72 haploinsufficiency in *C9orf72* ALS pathology.

### Knocking out *C9orf72* does not affect axonal transport or TDP-43 pathology but has a minor impact on cell survival

**Fig. 6:**
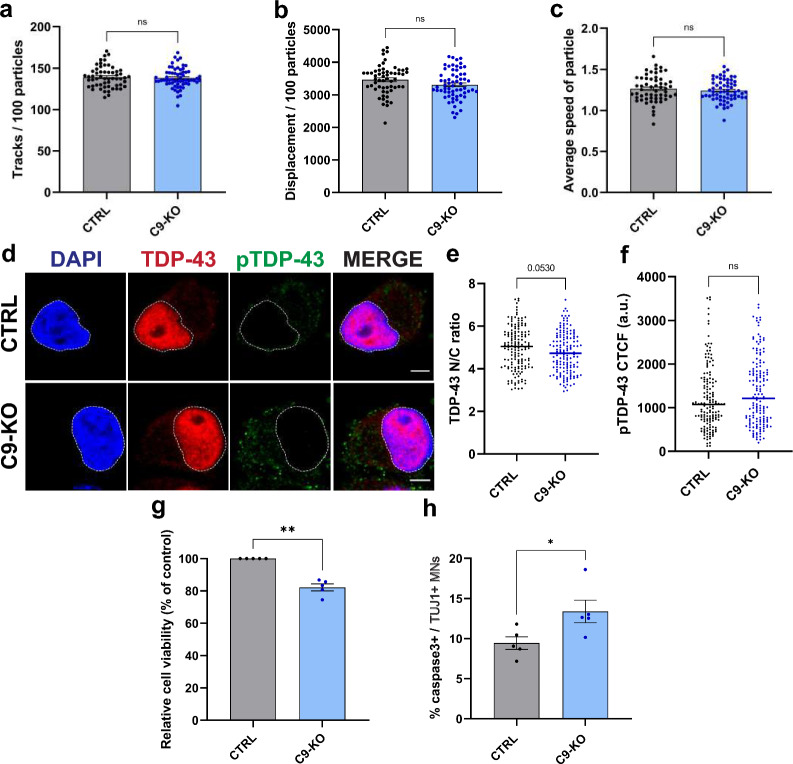
*C9orf72* KO MNs do not show with defects in lysosomal trafficking or TDP-43 localisation but do present with a reduction in cell viability. **A–c,** Quantification of vesicle transport along neurites in control and C9 KO iPSC-derived MNs loaded with Lysotracker Red. Total number of tracks per 100 particles (**a**), total displacement per 100 particles (**b**) and average speed of particles (**c**) were analyzed. Each dot represents a well that was analyzed (CTRL, n = 57; C9 KO, n = 63). **d** Immunocytochemistry (ICC) of aged, 60-day-old control and C9 KO MNs stained for TDP-43 and phosphorylated TDP-43 (pTDP-43). The presence of a typical neuronal morphology (which includes a soma and elongated neurites was used to select the MNs for analysis. Scale bar = 10 µm. **e**, **f** Quantifications of the nuclear vs cytoplasmic (N/C) TDP-43 ratio (**e**) and the corrected total cell fluorescence (CTCF) of pTDP-43 (**f**) from the ICC images shown in (**d**). Each dot represents a cell that was measured (CTRL, n = 153; C9 KO, n = 153). **g** Quantification of the relative cell viability in 60-day-old C9 KO MNs relative to its isogenic control line as measured by the CellTiter-Glo® assay. For each independent differentiation, at least 6 technical replicates of 5000 cells were plated in 96-well plates and the average intensity of the C9 KO line was normalized to that of its isogenic control line; each dot represents one biological replicate. **h** Quantification of the percentage of apoptotic cells staining positive for cleaved caspase-3 in the TUJ1-positive 60-day-old MN population. Each dot represents one biological replicate in which between 171 and 410 TUJ1-positive cells were scored for cleaved caspase-3 staining. Data represent mean ± SEM; data are pooled from five independent differentiations. Statistical significance was assessed by unpaired t-test (**a**–**c**, **e, f**, **h**) or one sample *t*-test (**g**); *p < 0.05, **p < 0.01

Although we could not detect reduced levels of C9orf72 protein, we wanted to test whether part of the phenotype we observed was related to C9orf72 loss-of-function. Therefore, we conducted a more in-depth investigation into the effect of *C9orf72* loss-of-function on our phenotypes by utilizing a homozygous *C9orf72* knockout iPSC line (C9-KO) (Additional file 1: Fig. S6a-e). Following differentiation of the C9-KO and control iPSC lines into MNs, we assessed the movement of Lysotracker Red-labeled vesicles and found no abnormalities in their motility (Fig. 6a–c). Additionally, we observed no significant differences in the subcellular localization or pathological phosphorylation of TDP-43 in 40-day-old (Additional file 1: Fig. S7a–c) or aged 60-day-old C9-KO iPSC-derived MNs (Fig. 6d–f). However, knocking out *C9orf72* did have an impact on cellular health as it reduced the cell viability of aged 60-day-old iPSC-derived MNs (Fig. 6g, h), but not of 40-day-old neurons (Additional file 1: Fig. S7d, e).

### Lysosome number, but not morphology, is altered in C9-KO MNs

**Fig. 7:**
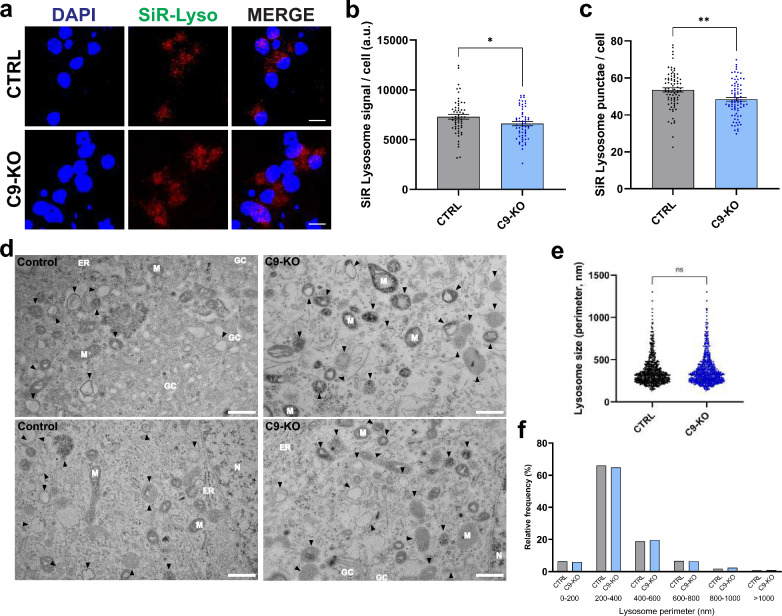
Knockout of C9orf72 seems to negatively affect lysosome number, but does not affect lysosome morphology. **a** Fluorescent microscopy images of 40-day-old C9 KO and isogenic control MNs labeled with SiR-Lysosome, a dye that selectively labels Cathepsin D-positive lysosomes. Scale bar = 50 µm. **b**, **c** Quantification of the SiR-Lysosome puncta number (**b**) and fluorescent intensity per cell (**c**) from the images shown in (**a**); each dot represents one confocal image that was analyzed (n = 60 for all conditions in (**b**) and n = 80 for all conditions in (**c**)). **d** Representative TEM images from 40-day old MNs used to quantify the size of lysosomes/late endosome (indicated by black arrowheads). Mitochondria (M), Golgi complex (GC), Nucleus (N) and the endoplasmic reticulum (ER) are marked on the images. **e**, **f** Relative frequency distribution (**e**) and quantification (**f**) of the lysosomal circumference measured from TEM images as shown in (**d**); each dot represents a lysosome that was measured (CTRL, n = 692; C9 KO, n = 765). Data represent mean ± SEM; data are pooled from three-four independent differentiations. Statistical significance was assessed by unpaired *t*-test (**b**, **c**) or Mann–Whitney *U* test (**e**); *p < 0.05, **p < 0.01

The role of *C9orf72* haploinsufficiency in *C9orf72* ALS/FTD and its broader physiological function in neurons are still subjects of debate. While C9orf72 clearly has a role in endolysosomal homeostasis, the exact mechanism and target organelles of C9orf72 differ between studies [12, 19, 69–74]. While most evidence suggests an interaction between C9orf72 and early endosomes, as well as a role in vesicle trafficking, lysosomes appear to be the most affected organelle population in both *C9orf72* patient and *C9orf72* knockout MNs [12, 19]. To further explore the possibility that loss of C9orf72 alone can induce lysosomal defects, we examined lysosomal number, morphology, and localization in C9-KO iPSC-derived MNs. We observed no differences in lysosomal localization (results not shown), but there was a modest yet significant reduction in mature, CTSD-positive lysosomes as measured by the SiR-Lysosome dye (Fig. 7a–c). However, examination of endolysosomal morphology using TEM revealed no signs of lysosomal enlargement in C9-KO iPSC-derived MNs (Fig. 7d–f). As the lysosomal defects observed in C9-KO MNs are less severe than those observed in *C9orf72* patient MNs, our data suggests that while *C9orf72* haploinsufficiency impacts lysosomal health, it is unlikely to be the primary factor contributing to neuronal lysosomal dysfunctions in *C9orf72* ALS/FTD. Additionally, *C9orf72* toxic GOF mechanisms may also play a role in disrupting lysosomal homeostasis.

### C9-KO MNs don’t show inhibition of autophagic flux but C9-KO causes an impairment of endosomal maturation

**Fig. 8:**
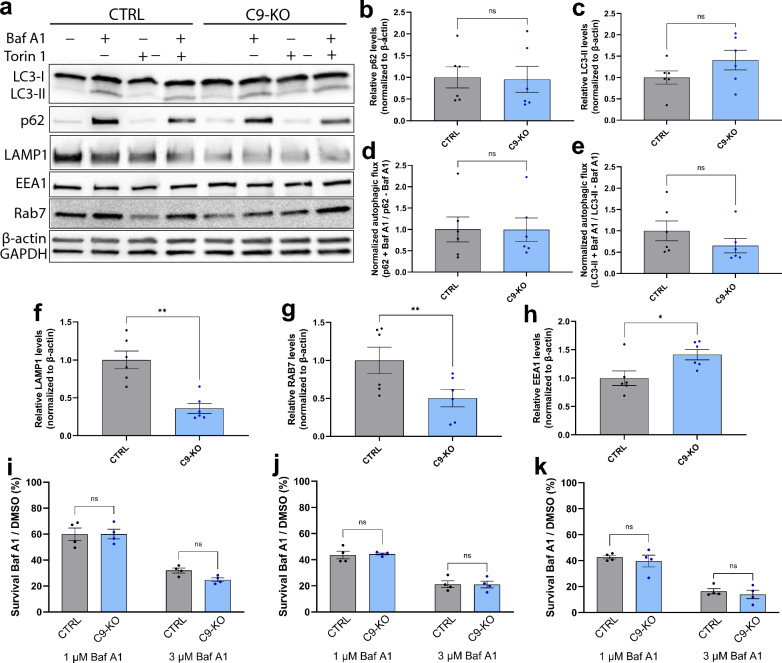
Endosome maturation, but not autophagy is impaired in C9 KO MNs.** a** Representative Western blot of iPSC-derived MNs treated with either DMSO, the autophagy inhibitor Bafilomycin A1 (Baf A1), the autophagy inducer Torin 1 or a combination of the latter, immunostained for LC3-I and LC3-II, p62, LAMP1, EEA1, Rab7 and β-actin/GAPDH, used to measure levels of autophagosomes, aggregates, lysosomes, early endosomes, late endosomes or for protein normalization respectively. **b**, **c** Quantifications of the Western blot shown in (**a**), measuring the relative levels of p62 (**b**) and the ratio of LC3-II/LC3-I (**c**); each dot represents one biological replicate. **d**, **e** Quantifications of the Western blot shown in (**a**), measuring the levels of p62 (**d**) or LC3-II (**e**) after treatment with Baf A1 and dividing those with the levels of either p62 or LC3-II without treatment; each dot represents one biological replicate. **f–h** Quantifications of the Western blot shown in (**a**), measuring the basal relative levels of LAMP1 (**f**), Rab7 (**g**) and EEA1 (**h**); each dot represents one biological replicate. **i–k** Quantification of the survival rate after 24 h (**i**), 48 h (**j**) or 72 h (**k**) treatment with Baf A1. The survival rate of iPSC-derived MNs is calculated by normalizing the CellTiter-Glo® signal of Baf A1-treated cells to DMSO control-treated wells; each dot represents the average of one biological replicate. Data represent mean ± SEM; data are pooled from six (**b–h**) or four (**i–k**) independent differentiations. Statistical significance was assessed by unpaired t-test (**b**–**h**) or one-way ANOVA and Tukey’s multiple comparison test (**i**–**k**); *p < 0.05, **p < 0.01

As we observed a small reduction in mature lysosomes upon *C9orf72* knockout, we further investigated basal autophagy and potential alterations in autophagic flux. Mature control and C9-KO iPSC-derived MNs were treated with DMSO vehicle, Baf A1, Torin 1 or a combination of both autophagy-modulating drugs (Fig. 8a). Treatments with Torin 1 revealed no apparent defects in autophagy induction (results not shown). Analysis of p62 levels (Fig. 8b), LC3-II/LC3-I ratio (Fig. 8c) and autophagic flux (Fig. 8d, e) showed no defects in untreated cells while we observed a significant decrease in LAMP1-levels in C9-KO cells (Fig. 8f). Given the involvement of C9orf72 in the endolysosomal pathway, we further validated the levels of Rab7 and EEA1, which serve as markers of late endosomes and early endosomes, respectively, in untreated cells (Fig. 8g, h). While Rab7 levels were also significantly downregulated in C9-KO neurons, EEA1 levels were significantly upregulated. These results indicate impaired endosomal maturation in MNs upon loss of C9orf72. Similar to the experiments performed in *C9orf72* patient iPSC-derived MNs (Fig. 5k–n), we investigated MN survival under autophagic stress induced by Baf A1. Interestingly, no differences in survival could be detected in any of the tested conditions upon depletion of C9orf72 (Fig. 8i–k). Consistently, treatment with another autophagy inhibitor, Lys05, also failed to induce survival differences in C9-KO iPSC-derived MNs (Additional file 1: Fig. S8b), while the survival rates of isogenic controls cells were comparable between all experiments (Additional file 1: Fig. S8c, d).

#### TBK1 is abnormally phosphorylated in *C9orf72* MNs

**Fig. 9:**
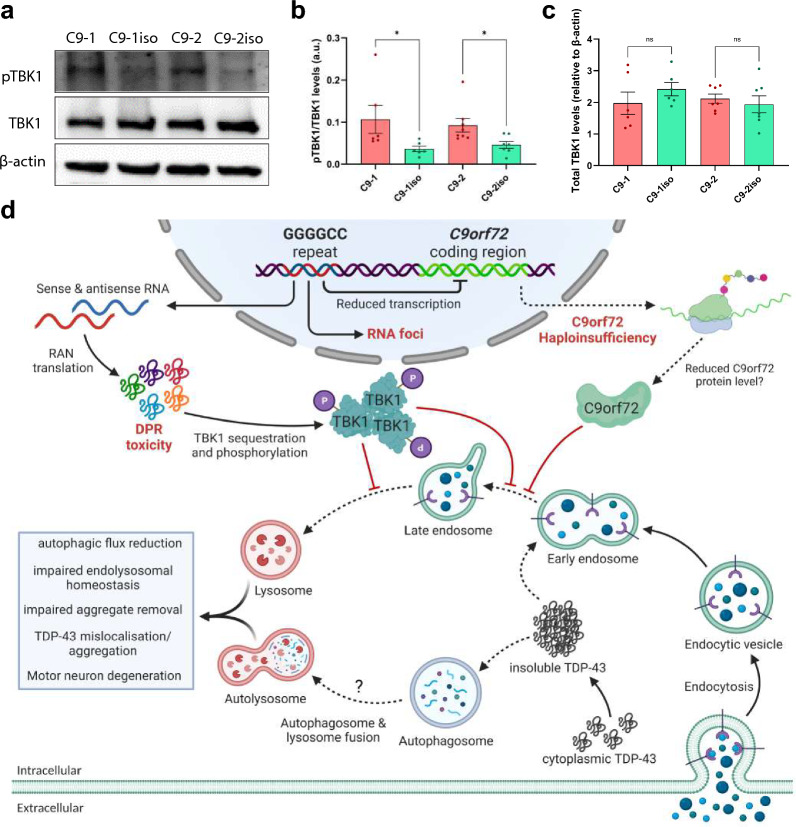
Phosphorylation of TBK1 at S172 is increased in *C9orf72* MNs.** a**, Representative Western blot of iPSC-derived MNs immunostained for pTBK (S172), TBK1 and β-actin. **b**, **c** Quantifications of the Western blot shown in (**a**), measuring the relative phosphorylation of TBK1 (**b**) and the total levels of TBK1 (**c**); each dot represents one biological replicate. **d**, Summary of the results found in this study: toxic GOF mechanisms in *C9orf72*-ALS cause impairment of (endo)lysosomal homeostasis, at least in part mediated by alteration of TBK1 signalling, while C9orf72 LOF mainly influences endosome maturation

A recent study by Shao et al*.* shed light on the interplay between *C9orf72*, TANK-binding kinase 1 (*TBK1*), and *TDP-43*, which are well-known ALS/FTD-associated genes [75]. The study revealed that aggregation of poly(GA) leads to the sequestration of TBK1 into inclusions, resulting in its transautophosphorylation [76, 77]. In light of these findings, we used immunoblot analysis to determine if TBK1 was also abnormally (hyper)phosphorylated in our *C9orf72* iPSC-derived MNs. Interestingly, while total TBK1 levels remained unaltered in lysates of 40-day-old patient iPSC-derived MNs and isogenic controls, *C9orf72* patient MNs exhibited an increased ratio of S172 phosphorylated TBK1 relative to total TBK1 (Fig. 9a–c). In summary, although we did not observe fully developed pathological aggregates containing pTBK1 in 40-day-old iPSC-derived MNs, the altered phosphorylation of TBK1 suggests perturbed TBK1 signaling in *C9orf72* patient MNs.

## Discussion

In this study, we used *C9orf72* patient-derived and *C9orf72* knockout hiPSC-derived MNs to investigate the early functional consequences of *C9orf72* HRE toxic gain-of-function mechanisms and *C9orf72* haploinsufficiency on neuronal health. We especially focused on exploring the impact on the autophagy and endolysosomal system, given the numerous genes associated with NDs have been linked to the endosome-lysosome network in recent years [8, 26, 28, 29]. Notable examples include ALS/FTD-linked variants in genes like *SQSTM1*/*p62*, *TBK1*, *C9orf72*, *OPTN*, *FIG4*, *ALS2*, *CHMP2B*, *FIG4* as well as FTD-causing mutations in *TMEM106* or *GRN* [8, 26, 28, 29]. Additionally, recent studies have discovered that the induction of endosomal abnormalities, either chemically or genetically, is sufficient to induce TDP-43 pathology [75]. This finding is not surprising, considering that TDP-43 turnover is partly dependent on endocytosis and autophagy [78, 79]. Our results demonstrate that MNs carrying the *C9orf72* HRE displayed defects in multiple aspects of the autophagy-lysosome pathway. These defects include dysregulated lysosomal transport and homeostasis, alterations in (endo)lysosomal morphology, decreased autophagic flux, and the accumulation of TDP-43 CTFs and p62 puncta. It is worth noting that these autophagy-lysosome abnormalities coincided with cytoplasmic mislocalization of TDP-43 and a decrease in neuronal cell viability. Interestingly, while the aforementioned ALS-related phenotypes became apparent upon aging of our hiPSC-derived MNs, lysosomal defects were already present at earlier stages, suggesting their potential involvement in disease onset or progression and emphasizing their clinical relevance.

During the early stages of our study, another research group working on *C9orf72* reported impaired axonal transport of the lysosomes [38]. Given the huge variability and lack of reproducibility among different research groups when working with hiPSCs, we were pleased to confirm the lysosomal axonal transport phenotype in our cellular models. To further validate our findings, we assessed ALS-related phenotypes in mature 40-day-old and more aged 60-day-old MNs, and we were able to recapitulate the reported TDP-43 mislocalization observed by other research groups [58, 79–83]. Additionally, we also found an age-related increase in TDP-43 phosphorylation and a decrease in cell viability of our iPSC-derived MNs. These results indicate that the presence or absence of certain ALS-related phenotypes during hiPSCs-derived MN differentiation may stem from variations in the differentiation protocols and the relative “age” of the analyzed MN cultures. As lysosomal homeostasis heavily relies on axonal transport, we further investigated the functional consequences of this dysregulation in *C9orf72* hiPSC-derived MNs. Using multiple independent methods, we observed a marked decrease in mature (CTSD-positive) lysosomes. In addition, we found an increase in enlarged lysosomes and lysosome-related organelles. Although enlarged lysosomes mostly contain active cathepsins, they are known to have problems with acidification, display reduced motility and dynamics, and consequently fail to travel into the axon and carry out their cellular function effectively [84, 85].

At the functional level, the alterations in lysosomal biology were accompanied by the accumulation of p62 puncta and insoluble fragments of TDP-43, both full-length and C-terminal. Despite not being able to detect full-blown TDP-43 or p62 aggregates, recent evidence suggests that these inclusions, reported in *post-mortem* samples, likely represent end-stage features of ALS and might not be essential to cause toxicity [34, 86]. Increased levels of p62 and insoluble TDP-43 in patient iPSC-derived MNs imply the possibility of a dysregulated autophagic system in these cells [60, 79]. While the initiation of autophagy was not impacted in *C9orf72* MNs, we observed an increase in the LC3-II/LC3-I ratio and in basal levels of p62 while LAMP1 levels where reduced in untreated *C9orf72* MNs. Interestingly, LAMP1 levels where found to be increased in *post-mortem* tissue from C9orf72 ALS patient. This discrepancy can be explained by the relatively small proportion of MNs in brain or spinal cord lysate compared to the pure MNs cultures in vitro. Additionally, treatment of our cells with Baf A1 revealed decreased autophagic flux. Consistent with these findings, *C9orf72* MNs displayed increased vulnerability to autophagy inhibition, further supporting the aforementioned autophagic defects. These results align with a previous study and provide additional evidence for impairment of the autophagy pathway as a pathogenic mechanism in *C9orf72* ALS/FTD [12, 22, 51].

Despite observing reduced C9orf72 protein levels in *post-mortem* spinal cord and central cortex samples of *C9orf72* ALS patients, we did not detect *C9orf72* haploinsufficiency in *C9orf72* patient iPSC-derived MNs. It is still unknown whether the lack of *C9orf72* haploinsufficiency in our iPSC-derived MNs can be attributed to the iPSC-reprogramming method or to the MN differentiation method or whether accurately models and thus challenges the hypothesis of haploinsufficiency in MNs, which remains controversial in the field. The reduced levels in *post-mortem* tissues may originate from reduced expression levels in cell types other than MNs and from the loss of MNs as well. In fact, a recent large-scale iPSC-derived MN differentiation study also reported significant variability in C9orf72 expression levels, which could explain the lack of C9orf72 haploinsufficiency observed in our study [87]. In addition, a recent single nuclei profiling study showed that C9orf72 mRNA levels vary a lot between the different cell populations present in the brain and spinal cord and that they were reduced mainly in microglia rather that in motor neurons [88]. As a consequence, our results rather point towards dysfunctions of autophagy in a later stage of the pathway (i.e., autophagosome-lysosome fusion or autolysosome degradation) caused by toxic HRE gain-of-function mechanisms although the possibility remains that reduced C9orf72 levels might negatively regulate autophagy initiation. In addition, C9orf72 haploinsufficiency might have a profound impact in other cell types such as microglia that show deficits in lysosomal transcriptional pathways and in that way directly influence motor neuron health [69, 88].

Overall, our findings demonstrate that defects in axonal trafficking, lysosomal homeostasis, and autophagy are already present in 40-day-old iPSC-derived MNs, preceding the onset of TDP-43 pathology and signs of neuronal degeneration that become apparent as the cells age. To contribute to understanding of C9orf72 haploinsufficiency, we assessed some of our phenotypes in *C9orf72* patient neurons in C9-KO iPSC-derived MNs. Surprisingly, we found little to no effects of C9orf72 protein loss on lysosomal transport or morphology, TDP-43 pathology or autophagy in neurons. However, this does not exclude a more dramatic impact of reduced C9orf72 levels in other cell types, such as microglia [89]. Nevertheless, we did observe small defects in neuronal survival and lysosome number upon C9orf72 knockout. Most research focusing on the physiological role of C9orf72 suggest its involvement in endolysosomal homeostasis and trafficking [19]. Interestingly, our data indicate that endosomal maturation is clearly impaired upon loss of C9orf72, further corroborating the implication of the C9orf72 protein in endolysosomal homeostasis. Therefore, while we cannot completely exclude the possibility of C9orf72 haploinsufficiency affecting autophagy, our results suggest that although C9orf72 clearly is an important player in the endolysosomal pathway and possibly other vesicle trafficking pathways, C9orf72 haploinsufficiency is highly unlikely to be the primary driving force behind the autophagic defects observed in MNs from C9orf72 ALS/FTD patients. In fact, most evidence points towards a form of synergistic pathogenesis in C9-ALS/FTD, primarily involving the toxic gain-of-function mechanisms of HRE, with C9orf72 haploinsufficiency making only a minor contribution.

Last but not least, we also observed an increased ratio of S172 phosphorylated TBK1 relative to total TBK1 in *C9orf72* patient MNs. Although we did not observe pathological aggregates of TBK1 in our 40-day-old MN cultures, hyperphosphorylation of TBK1 likely represents an early pathological event in the disease cascade. While this phosphorylation activates TBK1, its sequestration into cellular inclusions is believed to disrupt TBK1 activity and negatively impact endolysosomal maturation. As TBK1 is a crucial regulator of autophagic clearance of aggregates, its sequestration could initiate a viscous cycle that ultimately leads to the formation of full-blown aggregates (Fig. 9d) [77]. In fact, preliminary data from human MNs with reduced TBK1 activity also suggested a causal link between endolysosomal dysfunctions caused by TBK1 deficiency and TDP-43 pathology [46].

## Materials and methods

### Cell culture and motor neuron differentiation

The two human ALS patient-derived iPSC lines carrying a *C9orf72* HRE and their respective isogenic controls were previously described in [41], while the *C9orf72* KO line was was generated using SYNTHEGO multi-guide sgRNA to delete a 130-base pair (bp) fragment in exon 2 of the *C9orf72* gene (sgRNA-1: TTGGGCTCCAAAGACAGAAC; sgRNA-2: TAGCAGCTACTTTTGCTTAC; sgRNA-3: CCGCCATCTCCAGCTGTTGC). Control iPSCs were detached by incubation with Accutase for 3–5 min at 37°C. Upon dissociation into single cells, the required number of cells (5 × 105 cells per reaction) were resuspended in Lonza P3 Nucleofector solution (20 µL/reaction, P3 Primary Cell 4D-Nucleofector Kit S, Lonza). Cells in P3 Nucleofector solution were then mixed with 5 µL of Ribonucleoproteins (RNPs) (7.5:1 sgRNA to Cas9 ratio, previously incubated 10' at 37°C) and transferred into Nucleocuvette strip and electroporated using of the 4D-Nucleofector System with X unit (program "CA-137", Lonza). Nucleofected iPSCs were plated in a Matrigel-coated well plate with mTeSR1 and Y-27632 (10 μM) (Tocris) and incubated overnight at 37°C, 5% CO2. Upon confluence, cells were dissociated and plated at low density for clonal selection (500 and 250 cells per plate surface area of 9.6 cm2). Multiple colonies were manually picked and screened for optimal bp deletion (130-bp) via PCR. To validate the gene editing, the positive colonies were Sanger sequenced. The biallelic bp deletion followed by nonhomologous end-joining event led to the formation of multiple STOP codons within the exon 2 of the *C9orf72* gene. As a consequence, the translation of the C9orf72 protein was prematurely terminated, and no protein was detected via immunoblot analysis as shown in Fig S6e.

The iPSCs were cultured in Essential 8™ medium (E8 flex medium) (Thermo Fisher Scientific) supplemented with 1% penicillin–streptomycin (Thermo Fisher Scientific) and incubated at 37°C and 5% CO_2_. Upon reaching 80–90% confluence, iPSCs were passaged using 0.5mM Promega™ EDTA (Thermo Fisher Scientific) diluted in Gibco™ Dulbecco’s Phosphate-Buffered Saline (DPBS—Thermo Fisher Scientific) and plated on a 6-well plate coated with Geltrex® LDEV-Free hESC-Qualified Reduced Growth Factor Basement Membrane Matrix (Geltrex—Thermo Fisher Scientific). Once a month, the MycoAlert™ Mycoplasma Detection Kit (Lonza, Catalog Number: LT07-318) was used to confirm the absence of mycoplasma contamination. The differentiation of iPSCs into MN was performed as previously described [36]. The Ethical Committee of UZ Leuven gave ethical approval for these experiments (S50354).

### Immunocytochemistry

Cells were cultured on a coverslip before being fixed for 20 min using 4% PFA and rinsed three times with DPBS. Blocking was performed at room temperature for 1h using 5% normal donkey serum (NDS, Sigma), and 0.1% Triton X-100 in DPBS. Primary antibodies were diluted in 2% NDS, and 0.1% Triton X-100 in DPBS and incubated overnight at 4°C. A list of all antibodies used and their dilution can be found in Additional file 2: Table S1. After three washes with DPBS, the cells were incubated with the appropriate Alexa Fluor™ secondary antibodies (1:2000 in DPBS, Life Technologies; Additional file 2: Table S1) for 2h at room temperature. Subsequently, the coverslips were washed twice, and cell nuclei were stained using the NucBlue Live ReadyProbes™ Reagent (Thermo Fisher Scientific) for 20 min. Coverslips were again washed three times with DPBS before being mounted on microscope slides using ProLong™ Gold antifade reagent (Invitrogen). Confocal images were acquired using a Leica SP8 DMI8 confocal microscope (20 × or 64 × objective) using excitation lines at 405, 488, 555 and 647 nm. The acquired images were processed using Fiji software.

### Transmission electron microscopy (TEM)

Cells grown on glass bottom dishes (MatTek) were subjected to a series of procedures. First, they were washed with PBS, then with 0.1 M sodium cacodylate buffer, followed by overnight fixation using 2.5% glutaraldehyde in 0.1 M sodium-cacodylate buffer at 4 °C. Subsequently, the fixed cells were post-fixed in 1% osmium tetroxide (2 h), washed with dH_2_O, and subjected to gradual dehydration through an ethanol series (50–100%). During the 70% ethanol step, the samples were stained with uranyl acetate for 30 min at 4 °C. Following dehydration, cells were infiltrated with resin (Agar 100)/ethanol mixtures. The next day, cells were infiltrated and embedded with 100% epoxy resin in inverted BEEM® capsules for two days (60 °C). Ultrathin sections of 50 nm thick were cut following the separation of polymerized cells from the glass bottom dishes (using a freeze–thaw approach). These sections were subsequently post-stained with 3% uranyl acetate in water (10 min) and Reynold’s lead citrate (2 min). Micrographs were taken in a JEOL JEM2100 (JEOL, Japan) at 80 kV. Experiments were conducted in triplicate, and a minimum of 30 cells per condition were quantified. Ultrastructural identification and quantification of lysosomes were done as described [90] using RADIUS (EMSIS GmbH) software.

### Western blot analysis

iPSCs and iPSC-derived MNs were harvested using StemPro™ Accutase™ (Thermo Fisher Scientific). Following gentle centrifugation at 0.3g for 3 min, the cell pellet was snap-frozen on dry ice or using liquid nitrogen and subsequently stored at -80°C or directly homogenized. For homogenization, the cell pellet was treated with cold RIPA buffer (Sigma) supplemented with a protease inhibitor cocktail (cOmplete™ EDTA-free, Sigma) and phosphatase inhibitors (PhosSTOP, Sigma). The protein concentration was determined using the Micro BCA™ Protein Assay Kit (Thermo Fisher Scientific). Thereafter, equal amounts of protein (10–50 µg) were mixed with Pierce™ Reducing sample buffer (Thermo Fisher Scientific) and boiled for 10 min at 95°C before being loaded onto a precast 4–20% Mini-PROTEAN® TGXTM gel (Bio-Rad). After separation for 60–90 min at 100V, the proteins were transferred to a nitrocellulose membrane (Trans-Blot® Turbo™ Mini 0.2 µm Nitrocellulose Transfer Pack) by means of a Trans-Blot® Turbo™ Transfer System (Bio-Rad). Before immunodetection, total protein levels on the membrane were quantified using the No-Stain™ Protein Labeling Reagent (Invitrogen) according to the manufacturer’s guidelines. The membranes were then blocked in 5% nonfat dry milk (Blotting-Grade Blocker, Bio-Rad) in Tris Buffered Saline solution with 0.1% tween (TBS-T) for 1 h at room temperature before overnight incubation with primary antibodies. A list of primary antibodies and their dilutions can be found in Additional file 2: Table S1. The following day, primary antibodies were removed, and the membranes were washed 3 times for 10 min with TBS-T before incubating with specific secondary antibodies conjugated with horseradish peroxidase (1/5000, Agilent Dako) for 1 h. Finally, protein detection was performed using enhanced chemiluminescence reagents (ECL substrate, Thermo Fisher Scientific) and the ImageQuant LAS 4000 biomolecular Imager (GE Healthcare). The ImageQuant TL software (version 7.0; GE Healthcare Life Sciences) was used to quantify band intensities and normalize them to No-Stain™ total protein or β-actin loading controls.

### Soluble-insoluble protein fractionation

Cellular fractionation to seperate the insoluble protein fraction was achieved by centrifuging 250–500 µg of lysates from iPSC-derived MNs at 4°C for 20 min at maximum speed (20,000g). The supernatant was collected as soluble fraction (that still contains soluble but dispersible proteins [91]) and the pellet was washed once with RIPA buffer supplemented with protease and phosphatase inhibitors before being centrifuged a second time using identical settings. The supernatant was carefully removed and 25–50 µL of 2 × Laemmli sample buffer (Bio-Rad) supplemented with 1% DTT (dithiothreitol, Promega) was added to the pellet. Finally, 10–20 µg of the total protein fraction, soluble protein fraction and the entire insoluble protein fraction were analyzed using Western blot.

### Live cell imaging and axonal transport analysis

To measure the axonal transport of acidic organelles along microtubules, iPSC-derived MNs were allowed to mature until day 38 or 59 of the differentiation protocol. After washing the MNs with DPBS, the cells were incubated at room temperature for 30 min with LysoTracker™ RED DND-99 (200nM, Thermo Fisher Scientific) diluted in MN maturation medium. Subsequently, the media was replaced with Hibernate A Low Fluorescence medium (Brainbits) and image acquisition took place on the Operetta CLS™ High-Content Analysis System (PerkinElmer) at LiMoNe (VIB-KU Leuven). A total of 200 images were captured at 1s intervals using a 40× water immersion lens, and a 555 nm laser was used to excite the LysoTracker dye. Using Fiji, these 200 images were combined into a stack/video and time/distance kymographs were created for every individual axon. From these kymographs, the number of moving organelles was extracted as they can be discerned as tilted lines, whereas the stationary particles appear as vertical lines. Using these kymographs we could also detect pausing/stopping of organelles as this results in a short/long conversion of the tilted line to a vertical one. Next, using the trackmate plugin [92], the total amount of organelles were detected. In order to avoid unconscious bias, the analysis was carried out blinded.

### Lysotracker and DQ-Red BSA intensity measurements using flow cytometry

iPSC-derived MNs at DIV40 were washed once with DPBS and then incubated with 200nM of LysoTracker for 1 h at 37°C. Following a wash with DPBS, the cells were collected by treatment with Accutase. After centrifugation (5 min, 500g, 4°C) and removal of the supernatant, the cells were resuspended in 500 µL of 1% BSA solution and filtered prior to measuring Lysotracker fluorescence using a BD FACSymphony A5 flow cytometer recording a minimum of 50,000 positive events. Cells treated with bafilomycin A1 (100 nm) served as the control. Data analysis was performed using BD FACSDiva 8.0.1 software.

DQ-Red BSA was used as a substrate to evaluate the proteolytic activity of lysosomes. Briefly, iPSC-derived MNs at DIV40 were incubated with DQ-Red BSA (10µg/mL, Thermo Fisher Scientific) at 37°C for 1h. Following the incubation, single cells were collected by treatment with Accutase, centrifuged (5 min, 500g, 4°C) and resuspended in PBS (containing 1% BSA), and fluorescence was measured using a BD FACSymphony A5 flow cytometer, recording a minimum of 50,000 positive events were recorded. Cells treated with bafilomycin A1 (100 nm) were taken as a control. Data analysis was performed using BD FACSDiva 8.0.1 software.

### Cathepsin D activity assay

The proteolytic activity of Cathepsin D in C9orf72 or isogenic control iPSC-derived motor neurons was analyzed using the fluorometric Cathepsin D Activity Assay Kit (abcam). Samples were lysed in the CD lysis buffer and 100–200 ng of total protein was loaded in duplicates and analyzed as specified in the suppliers’ guidelines. In parallel, total protein samples were also analyzed using Western blot in order to estimate total cathepsin D protein levels.

### Cell viability assay

Cell viability of iPSC-derived MNs was assessed using the CellTiter-Glo® Luminescent Cell Viability Assay (Promega) according to the manufacturer’s guidelines. Briefly, MNs were grown in 96-well plates and at DIV40 or DIV60, an equal amount of CellTiter-Glo® reagent was added to the medium. The mixture was then shaken for 2 min to facilitate cell lysis followed by incubation of the plate for 10 min in the dark. Next, 100 µL of the reagent-media mixture was transferred to a white 96-well plate, and luminescence was recorded using the SpectraMax® iD3 microplate reader (Molecular Devices). Medium without cells was used to determine and correct for background luminescence.

### Survival assay of iPSC-derived MNs with autophagic stress

Following differentiation to motor neurons, varying concentrations of Baf A1 (1–3 µM) or Lys05 (3–30 µM) were added to the growth media on Day 37, Day 38 or Day 39. On Day 40, a complete medium change was performed, replacing the autophagic stressor or DMSO with fresh medium. Subsequently, cell survival was measured using the CellTiter-Glo® Luminescent Cell Viability assay as described above. The cell survival rate was determined by dividing the average luminescence intensity of wells treated with Baf A1 or Lys05 by the average intensity of control wells treated with DMSO.

### Autophagic flux assay

To measure autophagic flux, which represents the balance between autophagosome formation and degradation, we treated cells with either DMSO, 100nM Baf A1, 1µM Torin 1 or a combination of the latter two for 24 h. Thereafter, cells were snap-frozen or directly lysed and subjected to Western blot. By dividing the band intensities of either LC3-II or p62 in the Baf A1-treated cells with the intensities of the respective DMSO-treated cells, we estimated the autophagic flux occurring in the cells.

#### Human *post-mortem* brain and spinal cord samples

In accordance with the applicable laws in Belgium and upon written informed consent (UZ Leuven), brain and spinal cord tissue was collected. Ethical approval for the study was given by the Ethical Committee of UZ Leuven (S65097, S59292, S60803, Leuven, Belgium). Brain samples of 17 ALS cases (nine C9orf72 and eight sporadic) and seven non-neurodegenerative controls and spinal cord samples of (16 ALS cases (eight C9orf72 and eight sporadic) and eight non-neurodegenerative controls were included in this study (Additional file 3: Table S2). The diagnosis of ALS or FTD was based on clinical assessment according to the consensus criteria for ALS [93–95] and FTD [96, 97]. The *post-mortem* diagnosis of ALS and FTLD-TDP was confirmed through pathological evaluation of pTDP-43 pathology in both brain and spinal cord. After the autopsy, the right hemisphere was dissected in coronal planes of approx. 2 cm and frozen at -80°C. 50 mg of brain or spinal cord tissue was weighed and mechanically homogenized using a micropestle in 0.5 mL 2% SDS in TBS with Nuclease (Pierce™ Universal Nuclease, Thermo Fisher Scientific) and a cocktail of protease/phosphatase inhibitors (Halt, Thermo Fisher Scientific). The samples were then sonicated, followed by centrifugation at 14 000 g for 30 min. The resulting supernatant was used for further analysis, and protein concentrations were determined using the BCA Protein Assay Kit (Thermo Fisher Scientific).

#### Quantification of TDP-43 mislocalization

Using Fiji image analysis software, the nuclei of immunofluorescent-stained iPSC-derived motor neurons were selected in the DAPI channel. This was used to place corresponding regions of interest (ROIs) in the other channels in an unbiased manner. Cytoplasmic ROIs were captured for the selected nuclei in the p-TDP43 or Tuj1 channel. The intensity of TDP-43 in the nuclei and cytoplasm was measured as corrected total cell fluorescence (CTCF), which is calculated by subtracting the product of the mean fluorescence of the background and the area of the ROI from the integrated density. The data is presented as the ratio of nuclear to cytoplasmic intensity (N/C ratio), and each data point represents a single cell.

#### Quantification of p62 puncta

To analyze the number of p62 puncta in cells, the “Analyze Particles” function of Fiji image analysis software was used, with the "MaxEntropy" threshold option selected as previously described in [47]. In brief, the channel of interest (p62) was extracted from the confocal image, and the cell of interest was cropped out. The Image was then duplicated, and the "MaxEntropy" threshold was applied to the duplicated image. We then measured the “Area” and the “Mean gray value” and limited our measurement to our threshold while also redirecting the measurement to the original image. Finally, we used the “Analyze Particles” function from the “Analyze” tab to obtain size and intensity measurements from every single p62 puncta in the cell.

#### Statistics

Data was analyzed and visualized using GraphPad Prism 9.5.0. Statistical tests as well as sample numbers and biological replicates, are indicated in the respective figure legends.

### Supplementary Information

** Additional file 1**: **Figure S1**: Generation and characterization of iPSC-derived MNs from *C9orf72* ALS patients and isogenic controls. **a** Schematic representation of the protocol used to differentiate iPSCs to spinal motor neurons (MNs). Abbreviations: BDNF: brain-derived neurotrophic factor; CHIR: CHIR99021; CNTF: ciliary neurotrophic factor; DAPT: a γ-secretase inhibitor; GDNF: glial cell line-derived neurotrophic factor; iPSC: induced pluripotent stem cell; LDN: LDN-193189; NEP: neuroepithelial stem cell; NPC: neuronal progenitor cell; RA: retinoic acid; SAG: smoothened agonist; SB: SB 431542; Y: Y-27632. **b, c** Immunocytochemistry (ICC) of multiple (motor) neuron markers TUJ1 and ISL1 (**b**), ChAT and SMI32 (**c**) and DAPI in 40-day old *C9orf72* and isogenic control MNs. Scale bar = 50 µm. **d**–**f** Quantification of the ISL-positive (**d**), ChAT-positive (**e**), and SMI-32 positive (**f**) cells relative to the total DAPI-labeled cell count; each dot represents one biological replicate. Data represent mean ± SEM. Statistical significance was assessed by one-way ANOVA (**d**–**f**) and Tukey’s multiple comparison test (b, c, t); ns = not significant. **Figure S2**: C9orf72 MNs have defects in lysosomal function and display reduced levels of mature lysosomes. **a** Flow cytometry analysis graphs of MNs stained with Lysotracker Red. **b** Quantification of the relative Lysotracker Red fluorescence shown in (**a**). **c** Flow cytometry graphs of MNs treated with DQ-BSA which generates fluorescence upon proteolytic cleavage by lysosomes**. d** Quantification of the relative DQ-BSA fluorescence shown in (**c**). **e** Representative Western blot detecting pro-cathepsin D (CTSD) and mature CTSD. β-actin was used to normalize data. **f** Quantifications of the Western blot shown in (**e**), measuring the relative levels of mature CTSD. **g** Quantification of the relative QTSD enzyme activity as measured by a fluorometric CTSD activity assay kit. Data represent mean ± SEM. Statistical significance was assessed by Kruskal–Wallis test and Dunn’s multiple comparison test (**b**, **d**, **f**, **g**); *p < 0.05, **p < 0.01. **Figure S3**: Mature *C9orf72* MNs do not display mislocalization of TDP-43, nor increased phosphorylation of TDP-43 and only display a minor reduction in cell viability**. a** Immunocytochemistry (ICC) of 40-day old C9orf72 and isogenic control MNs stained for TDP-43 and phosphorylated TDP-43 (pTDP-43). The presence of a typical neuronal morphology (which includes a soma and elongated neurites was used to select the MNs for analysis. Scale bar = 10 µm. **b**, **c** Quantifications of the nuclear vs cytoplasmic (N/C) TDP-43 ratio (**b**) and the corrected total cell fluorescence (CTCF) of pTDP-43 (**c**) from the ICC images shown in (**a**). Each dot represents a cell that was measured (C9-1, n = 166; C9-1iso, n = 189; C9-2, n = 163; C9-2iso, n = 172). **d** Quantification of the relative cell viability in 40-day-old C9orf72 MNs relative to their isogenic controls as measured by the CellTiter-Glo® assay. For each independent differentiation, at least 6 technical replicates of 5000 cells were plated in 96-well plates the average intensity of each C9orf72 patient line was normalized to that of their respective isogenic control; each dot represents one biological replicate. **e**, Quantification of the percentage of apoptotic cells staining positive for cleaved caspase-3 in the TUJ1-positive 40-day-old MN population. Each dot represents one biological replicate in which between 107 and 224 TUJ1-positive cells were scored for cleaved caspase-3 staining. Data represent mean ± SEM; data are pooled from four-five independent differentiations. Statistical significance was assessed by Kruskal–Wallis test and Dunn’s multiple comparison test (**b**, **c**) or one-way ANOVA and Tukey’s multiple comparison test (**d**, **e**); *p < 0.05, ns: not significant. **Figure S4**: Signs of dysregulations in the autophagy-lysosome pathway are present in* post-mortem* brain and spinal cord tissue of ALS patients. **a** Representative Western blot detecting LC3-I and LC3-II, LAMP1, GAPDH and total protein, used to measure levels of autophagosomes, lysosomes or for protein normalization respectively in *post-mortem* human central cortex tissue lysates from C9orf72 ALS patients (C9orf72), ALS patients tested negative for the C9orf72 HRE (Non-C9 ALS) and healthy controls (Control). **b**, **c** Quantifications of the Western blot shown in (**a**), measuring the ratio of LC3-II/LC3-I (**b**) and relative levels of LAMP1 (**c**); (C9orf72, n = 9; Non-C9 ALS, n = 8; Control, n = 7). GAPDH or total protein was used to normalize data. **d,** Representative Western blot detecting LC3-I and LC3-II, p62, LAMP1, GAPDH and total protein, used to measure levels of autophagosomes, aggregates, lysosomes or for protein normalization respectively in *post-mortem* human cervicothoracic spinal cord tissue lysates from C9orf72 ALS patients (C9orf72), ALS patients tested negative for the C9orf72 HRE (Non-C9 ALS) and healthy controls (Control). **e**–**g**, Quantifications of the Western blot shown in (**d**), measuring the ratio of LC3-II/LC3-I (**e**), the relative levels of LAMP1 (**f**) and the relative levels of p62 (**g**); each dot represents one tissue sample (C9orf72, n = 8; Non-C9 ALS, n = 8; Control, n = 8). GAPDH or total protein was used to normalize data. Data represent mean ± SEM. Statistical significance was assessed by Kruskal–Wallis test and Dunn’s multiple comparison test (**b**, **c**, **e**–**g**); *p < 0.05. **Figure S5**: C9orf72 protein levels are downregulated in *post-mortem* brain and spinal cord samples, but not in *C9orf72* MNs. **a**–**c** Representative Western blots detecting C9orf72, GAPDH or β3-tubulin and total protein in *post-mortem* human central cortex (**a**) or human cervicothoracic spinal cord tissue lysates (**c**) from *C9orf72* ALS patients (C9orf72), ALS patients tested negative for the *C9orf72* HRE (Non-C9 ALS) and healthy controls (Control) and *C9orf72* patient iPSC-derived MNs and their respective isogenic controls (**c**). **d**–**f** Quantifications of the Western blots shown in (**a**–**c**), measuring the levels of C9orf72 in human central cortex (**d**), human cervicothoracic spinal cord (**e**) and *C9orf72* patient iPSC-derived MNs and their respective isogenic controls (**f**); each dot represents one tissue sample (C9orf72, n = 8–9; Non-C9 ALS, n = 8; Control, n = 7–8) or one independent differentiation. GAPDH, β3-tubulin or total protein was used to normalize data. Data represent mean ± SEM; data are pooled from twelve-thirteen (**f**) independent differentiations. Statistical significance was assessed by Kruskal–Wallis test and Dunn’s multiple comparison test (**d**, **e**) or one-way ANOVA and Tukey’s multiple comparison test (**f**); *p < 0.05. **Figure S6**: Generation of C9orf72 knockout line (C9-KO). **a** General overview of the C9-KO iPSC line generation. **b**, Schematic representation showing multi-guide RNAs targeting exon 2 of the *C9orf72* gene (upper panel). Sanger sequencing traces of homozygous C9-KO and CTRL lines spanning the cut side of guide 1 (lower panel) **c** PCR product showing 130 bp deletion in the C9-KO line. **d** The C9-KO iPSC line retains pluripotency features as assessed by immunostaining of OCT4, SOX2, and NANOG. Scale bar = 100 µm. **e,** Representative Western blots detecting C9orf72 and total protein levels in iPSCs (left) or 40-day-old MNs derived from these iPSCs (right). **Figure S7**: Mature C9-KO iPSC-derived MNs do not display mislocalization of TDP-43, nor increased phosphorylation of TDP-43 and only a minor reduction in cell viability.** a** Immunocytochemistry (ICC) images of 40-day-old control and C9 KO MNs stained for TDP-43 and phosphorylated TDP-43 (pTDP-43). The presence of a typical neuronal morphology (which includes a soma and elongated neurites was used to select the MNs for analysis. Scale bar = 10 µm. **b**, **c** Quantifications of the nuclear vs cytoplasmic (N/C) TDP-43 ratio (**b**) and the corrected total cell fluorescence (CTCF) of pTDP-43 (**c**) from the ICC images shown in (**a**). Each dot represents a cell that was measured (CTRL, n = 168; C9 KO, n = 173). **d** Quantification of the relative cell viability in 40-day-old C9 KO MNs relative to its isogenic control line as measured by the CellTiter-Glo® assay. For each independent differentiation, at least 6 technical replicates of 5000 cells were plated in 96-well plates and the average intensity of the C9 KO line was normalized to that of its isogenic control line; each dot represents one biological replicate. **e,** Quantification of the percentage of apoptotic cells staining positive for cleaved caspase-3 in the TUJ1-positive 40-day-old MN population. Each dot represents one biological replicate in which between 191 and 279 TUJ1-positive cells were scored for cleaved caspase-3 staining. Data represent mean ± SEM; data are pooled from four-five independent differentiations. Statistical significance was assessed by unpaired t-test (**b, c**, **e**) or one sample t-test (**d**); *p < 0.05, **p < 0.01. **Figure S8**: C9orf72 patient, but not C9-KO MNs display an increased vulnerability to autophagic stress caused by Lys05. **a, b** Quantifications of the survival rate of 40-day-old C9orf72 patient (**a**) or C9-KO (**b**) iPSC-derived MNs and their respective isogenic control after 24 h treatment with 3-30 µM of Lys05. The survival rate of iPSC-derived MNs is calculated by normalizing the CellTiter-Glo® signal of Lys05-treated cells to DMSO control-treated wells; each dot represents the average of one biological replicate. Data represent mean ± SEM; data are pooled from three-six (**a**) or four (**b**) independent differentiations. Statistical significance was assessed by one-way ANOVA and Tukey’s multiple comparison test (**a**, **b**); *p < 0.05, **p < 0.01, ***p < 0.001, ****p < 0.0001.

**Additional file 2**:** Table S1**. List of reagents and resources.

**Additional file 3**:** Table S2**. Clinical information of post-mortem samples.

## Data Availability

Microscopy data reported in this paper will be shared by the corresponding author upon reasonable request. Any additional information required to reanalyse the data reported in this paper is available from the corresponding author upon reasonable request.
