# Supplementary figures and images for: A toxic gain-of-function mechanism in C9orf72 ALS impairs the autophagy-lysosome pathway in neurons
Source: Acta Neuropathol Commun. 2023 Sep 18;11:151. doi: 10.1186/s40478-023-01648-0 (PMC10506245; doi:10.1186/s40478-023-01648-0)

**a**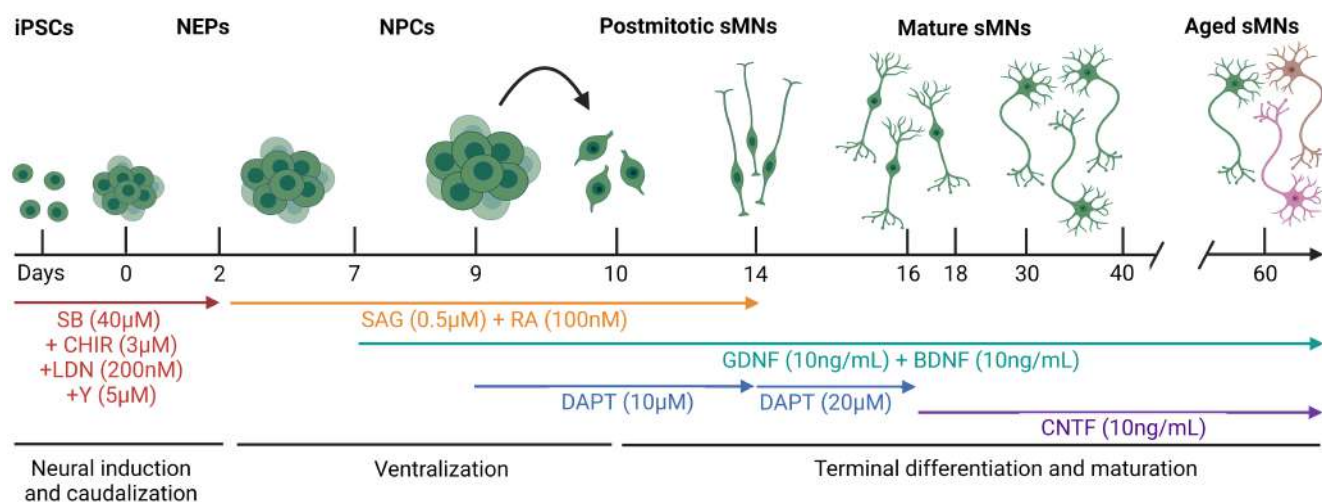**b**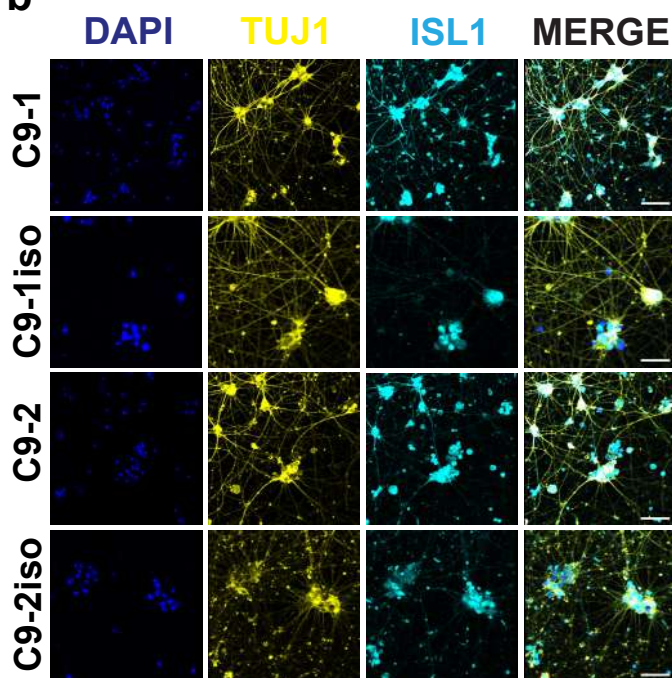**c**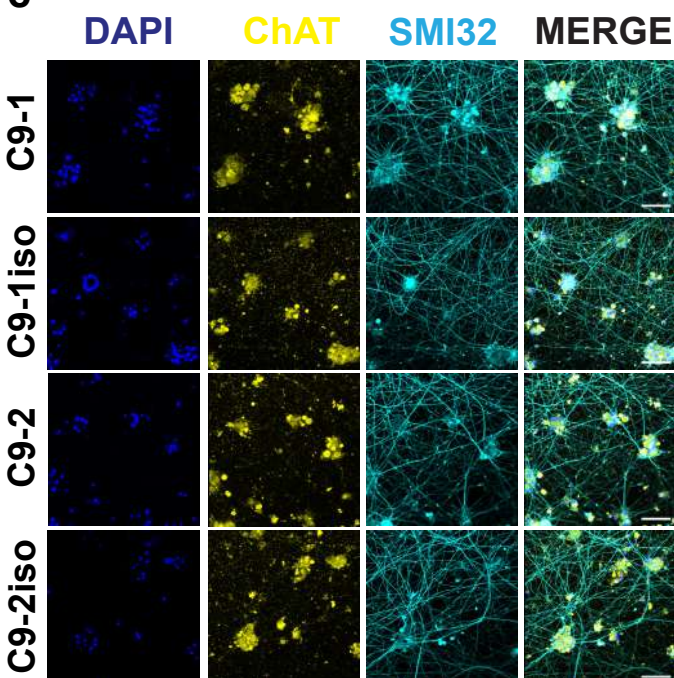**d**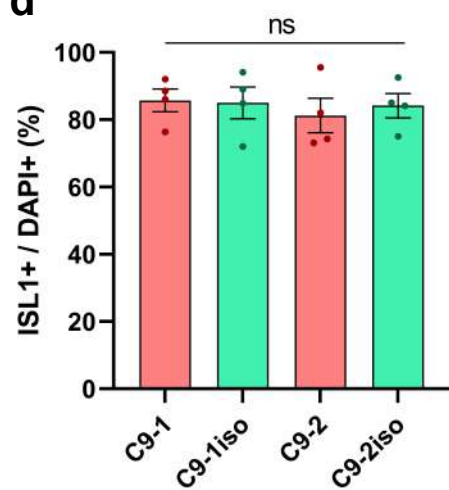**e**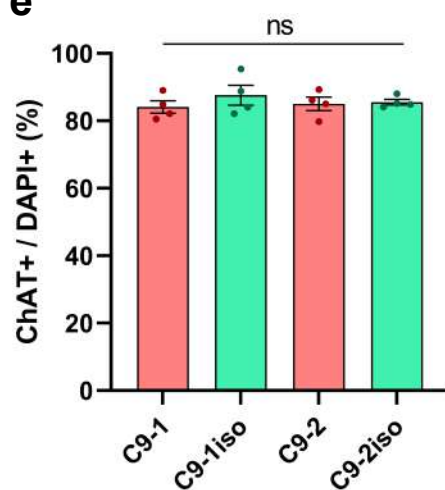**f**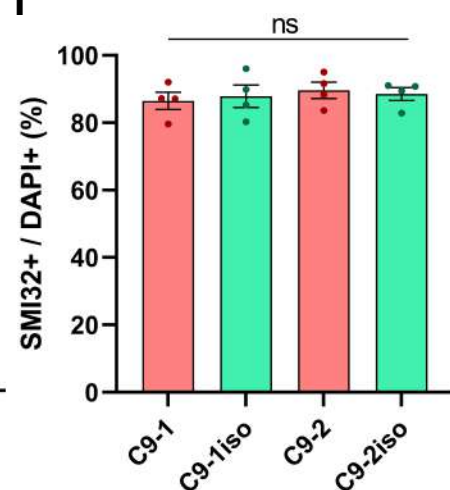

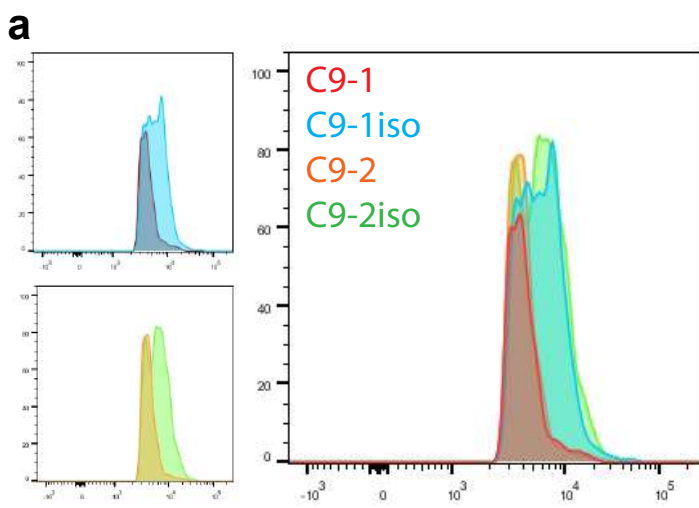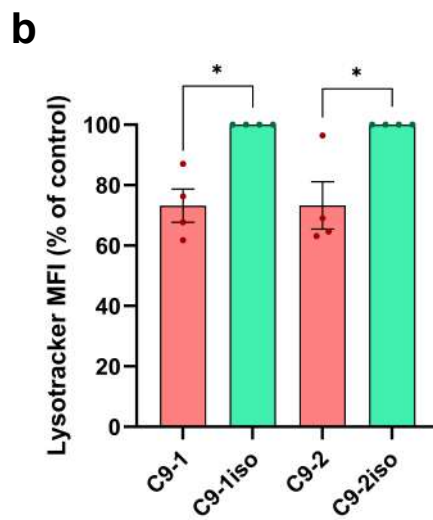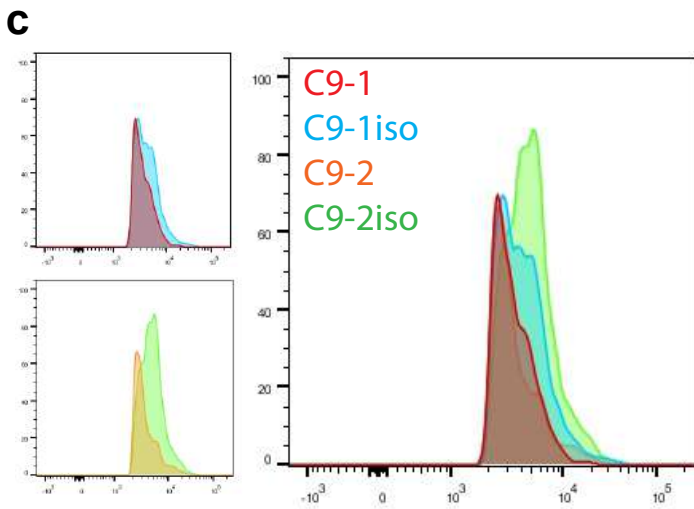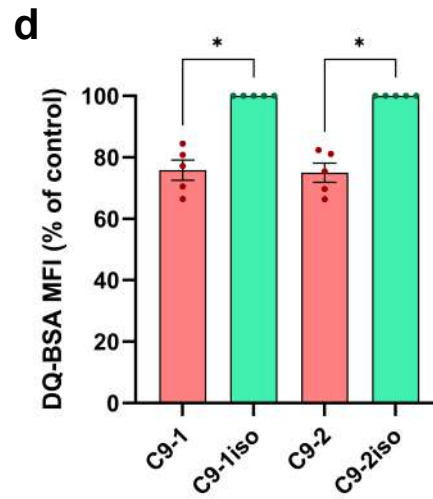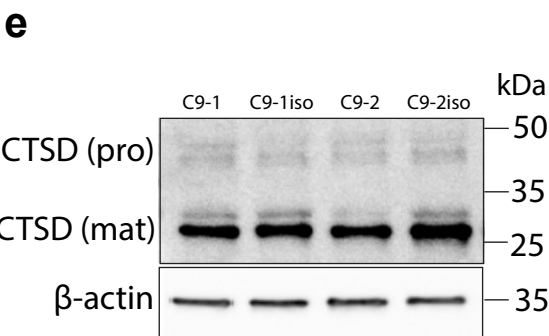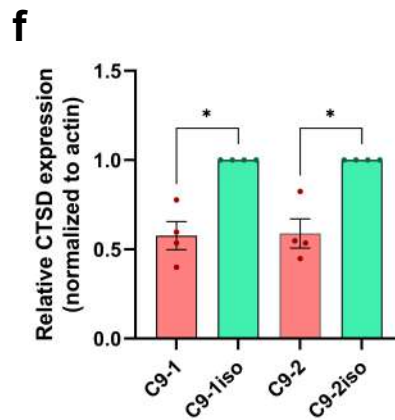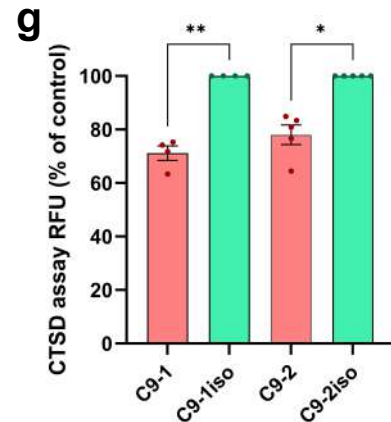

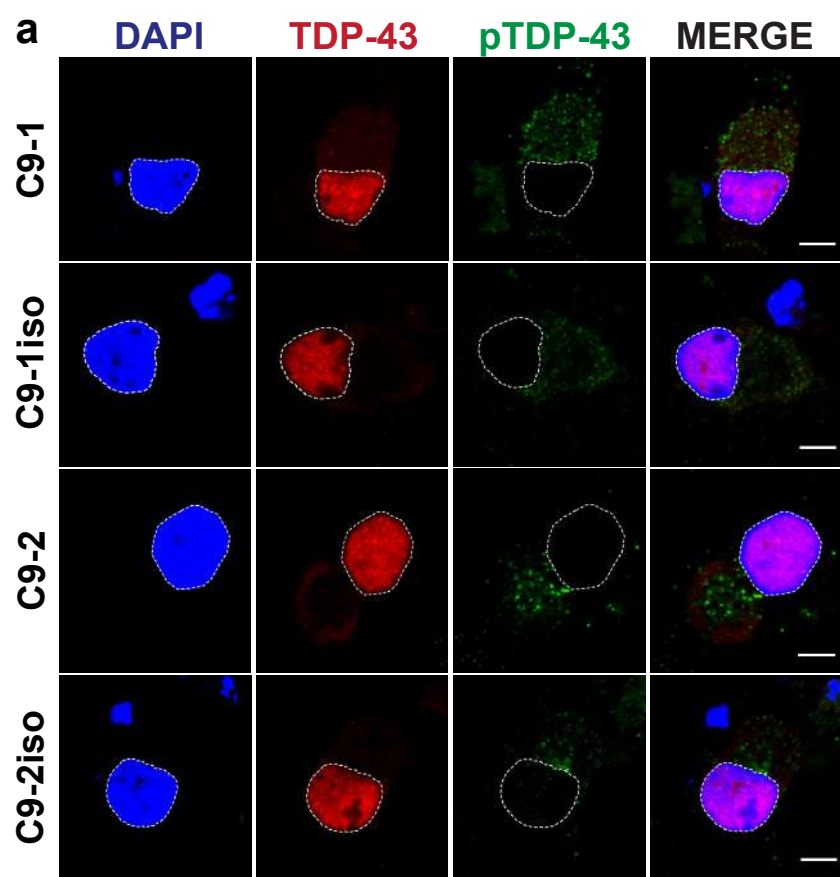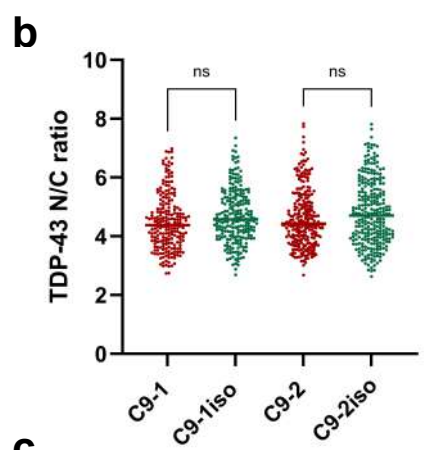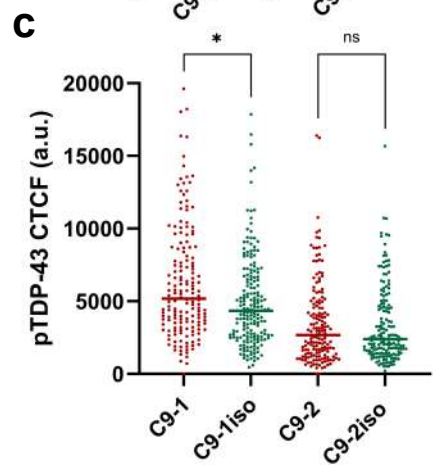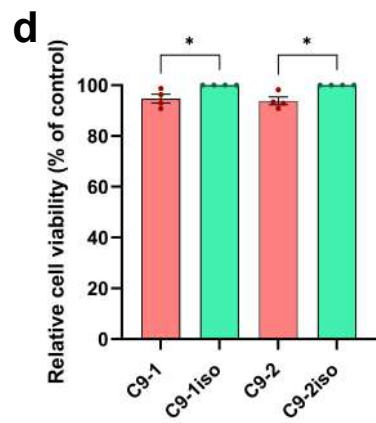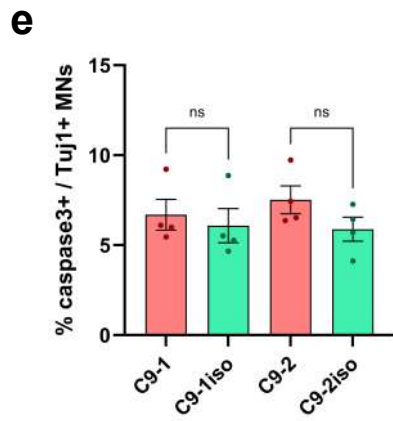

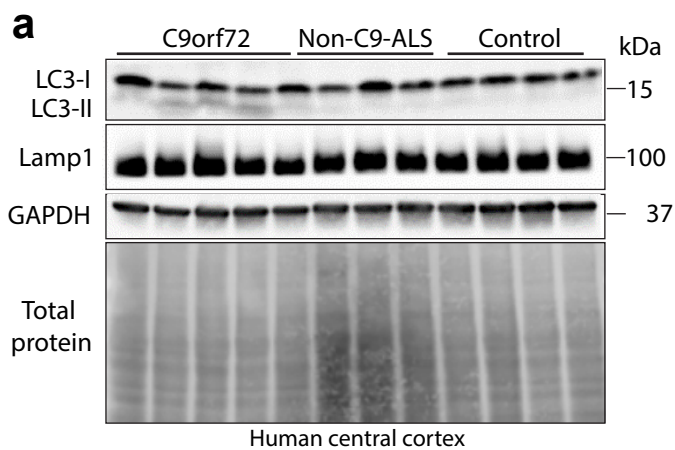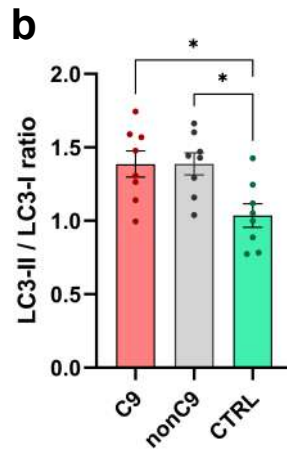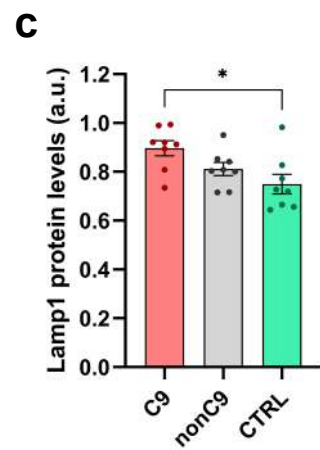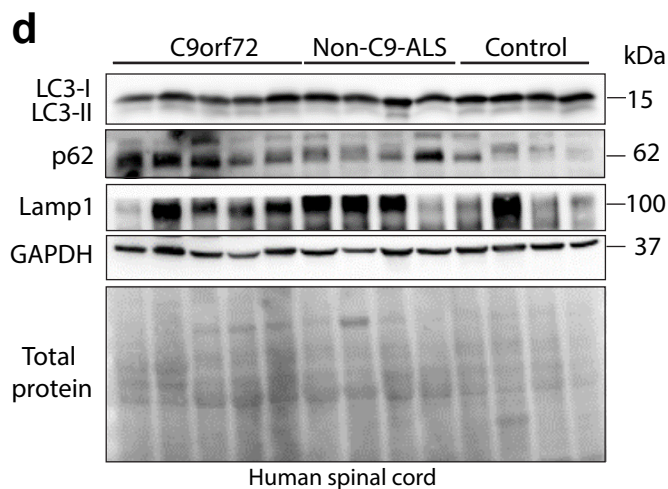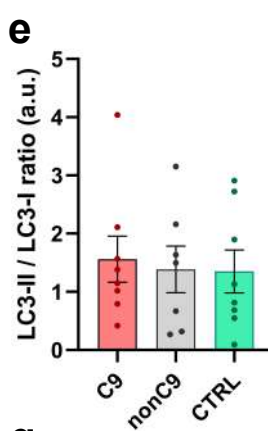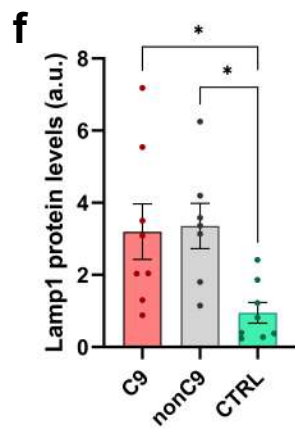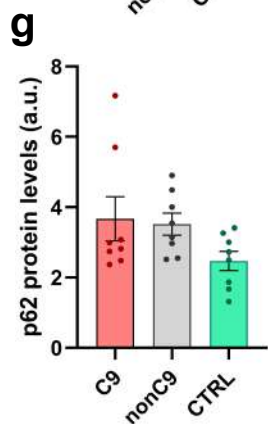

**a**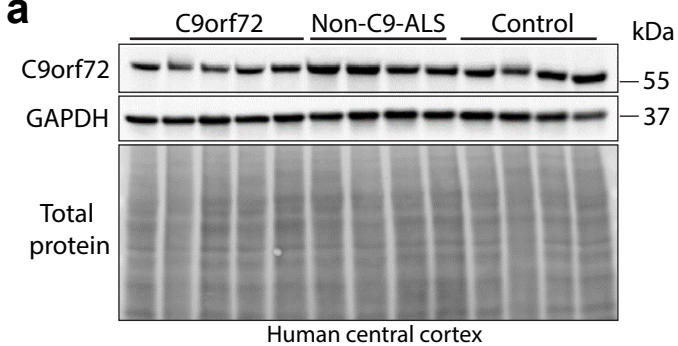**b**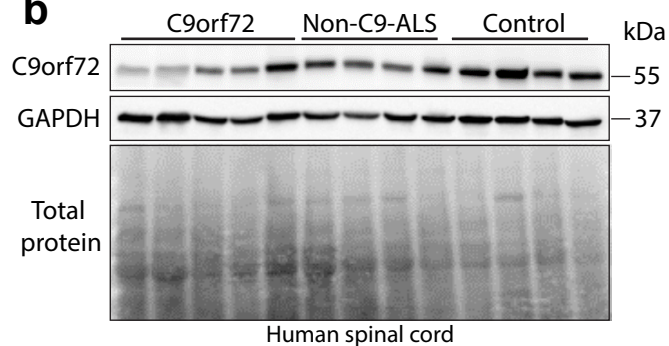**c**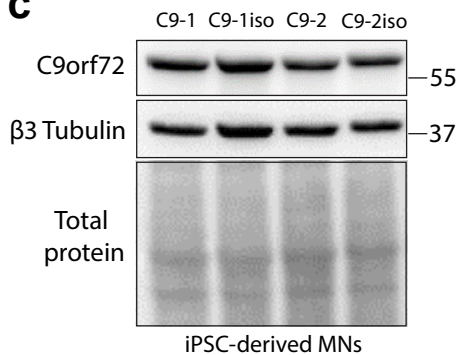**d**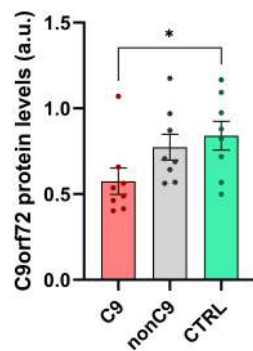**e**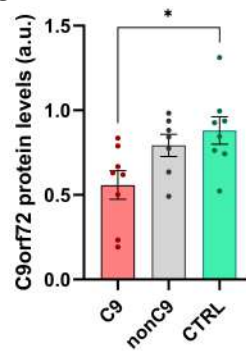**f**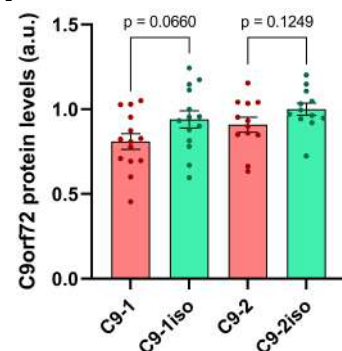

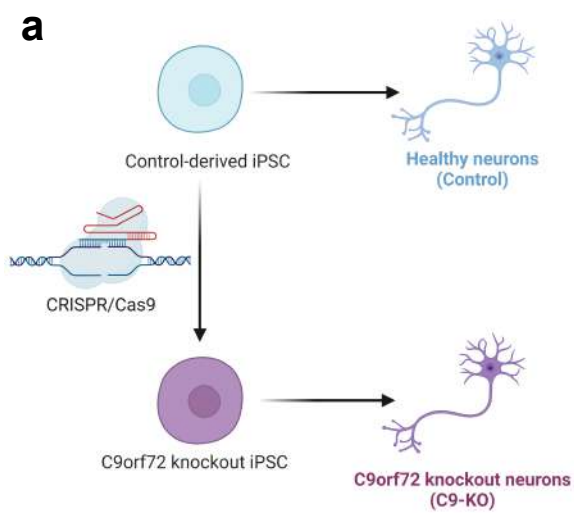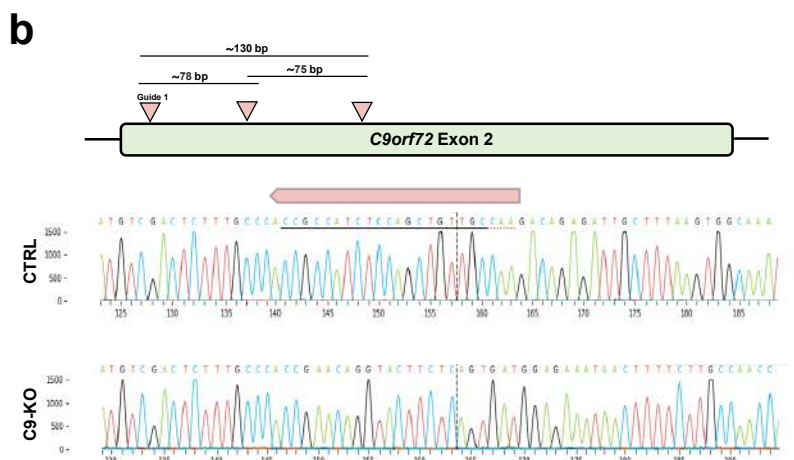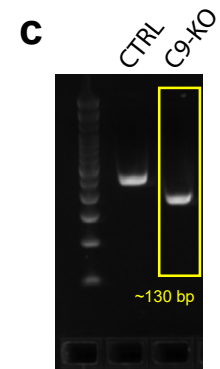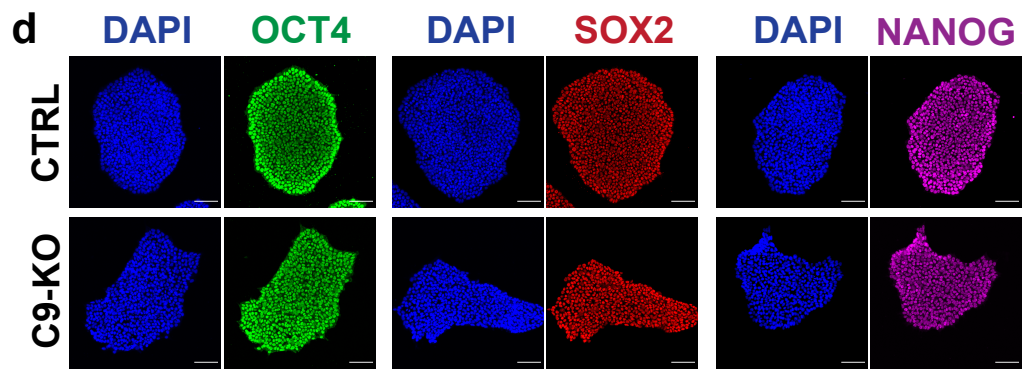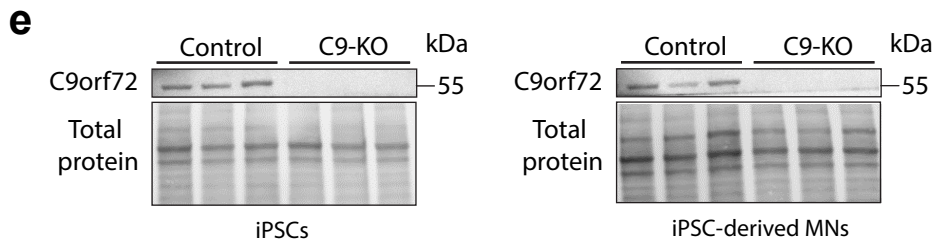

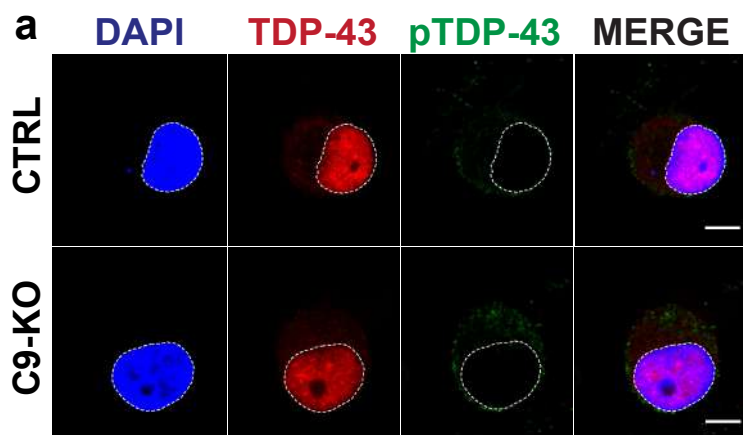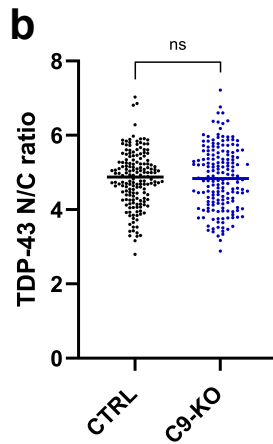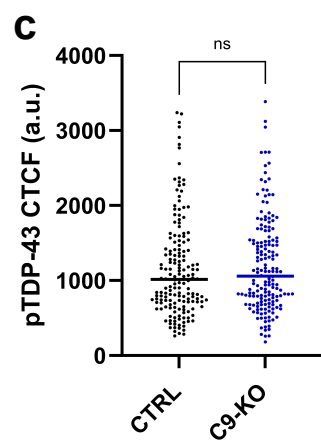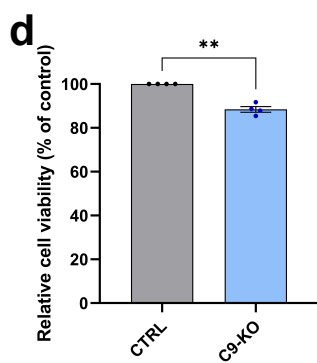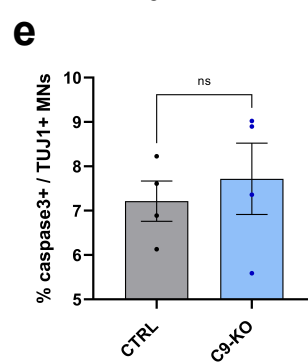

**a**

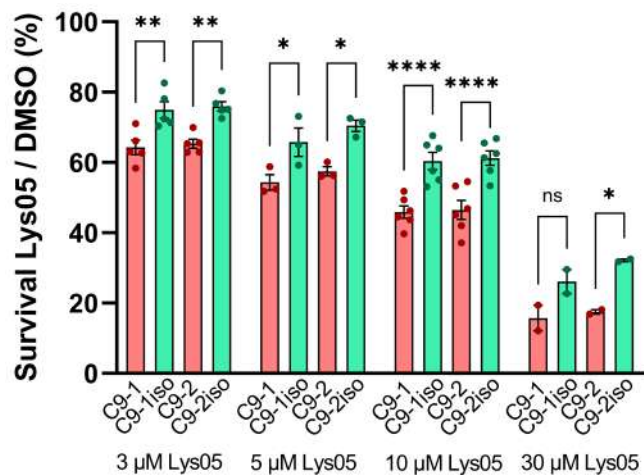

**C**

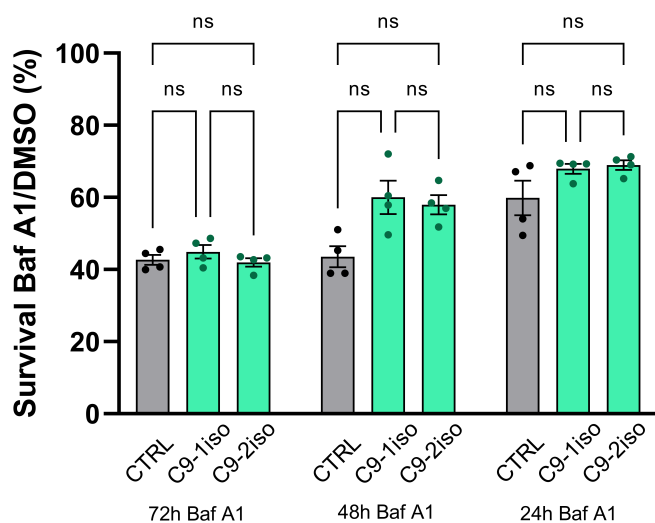

**b**

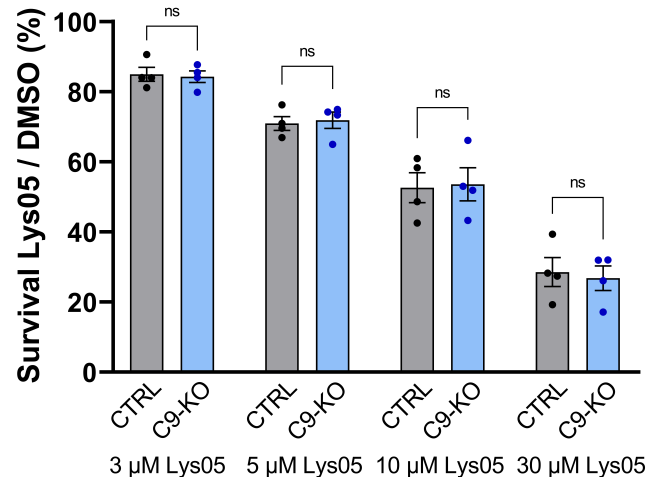

**d**

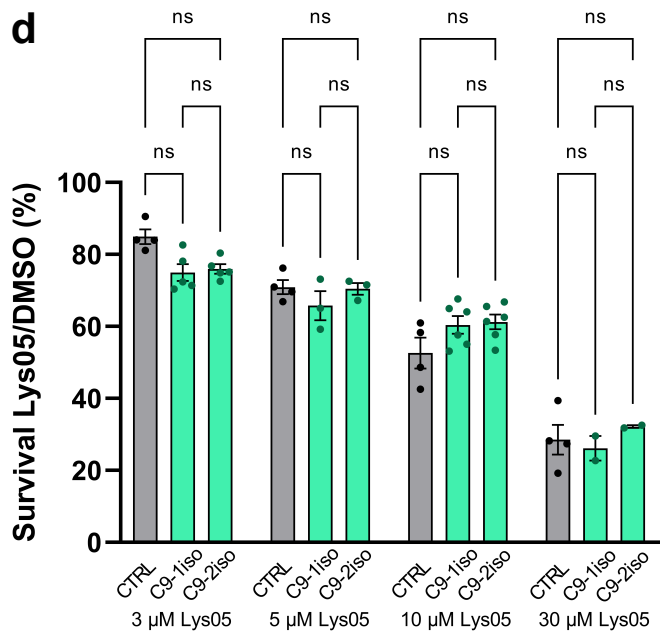

Supplement: Supplementary file 1 — Additional file 1: Figure S1: Generation and characterization of iPSC-derived MNs from C9orf72 ALS patients and isogenic controls. a Schematic representation of the protocol used to differentiate iPSCs to spinal motor neurons (MNs). Abbreviations: BDNF: brain-derived neurotrophic factor; CHIR: CHIR99021; CNTF: ciliary neurotrophic factor; DAPT: a γ-secretase inhibitor; GDNF: glial cell line-derived neurotrophic factor; iPSC: induced pluripotent stem cell; LDN: LDN-193189; NEP: neuroepithelial stem cell; NPC: neuronal progenitor cell; RA: retinoic acid; SAG: smoothened agonist; SB: SB 431542; Y: Y-27632. b, c Immunocytochemistry (ICC) of multiple (motor) neuron markers TUJ1 and ISL1 (b), ChAT and SMI32 (c) and DAPI in 40-day old C9orf72 and isogenic control MNs. Scale bar = 50 µm. d–f Quantification of the ISL-positive (d), ChAT-positive (e), and SMI-32 positive (f) cells relative to the total DAPI-labeled cell count; each dot represents one biological replicate. Data represent mean ± SEM. Statistical significance was assessed by one-way ANOVA (d–f) and Tukey’s multiple comparison test (b, c, t); ns = not significant. Figure S2: C9orf72 MNs have defects in lysosomal function and display reduced levels of mature lysosomes. a Flow cytometry analysis graphs of MNs stained with Lysotracker Red. b Quantification of the relative Lysotracker Red fluorescence shown in (a). c Flow cytometry graphs of MNs treated with DQ-BSA which generates fluorescence upon proteolytic cleavage by lysosomes. d Quantification of the relative DQ-BSA fluorescence shown in (c). e Representative Western blot detecting pro-cathepsin D (CTSD) and mature CTSD. β-actin was used to normalize data. f Quantifications of the Western blot shown in (e), measuring the relative levels of mature CTSD. g Quantification of the relative QTSD enzyme activity as measured by a fluorometric CTSD activity assay kit. Data represent mean ± SEM. Statistical significance was assessed by Kruskal–Wallis test and Dunn’s mult [file 40478_2023_1648_MOESM1_ESM.pdf]
